# Impact of trigger-day serum luteinizing hormone levels on embryo quality and pregnancy outcomes in overweight and obese women undergoing GnRH antagonist protocols: a retrospective cohort study

**DOI:** 10.3389/fendo.2026.1825688

**Published:** 2026-05-08

**Authors:** Danping Li, Wenhan Ju, Xina Zhen, Demin Lv, Wenli Yang, Shan Xiang, Fangting Duan

**Affiliations:** 1 First Clinical Medical College of Shandong University of Traditional Chinese Medicine, Jinan, China; 2 Guanghua Hospital Affiliated to Shanghai University of Traditional Chinese Medicine, Shanghai, China; 3 Qingdao Huangdao District Traditional Chinese Medicine Hospital, Qingdao, Shandong, China

**Keywords:** embryo quality, GnRH antagonist protocol, luteinizing hormone, overweight, pregnancy outcomes

## Abstract

**Introduction:**

Luteinizing hormone (LH) is critical for follicular development and IVF pregnancy outcomes, but evidence linking trigger-day serum LH levels to IVF-ET outcomes in overweight patients is scarce. This study investigated the impact of trigger-day LH levels on IVF-ET outcomes in overweight and obese women (BMI ≥ 25 kg/m^2^) undergoing flexible GnRH antagonist controlled ovarian stimulation (COS).

**Methods:**

Clinical data from 1,135 overweight and obese women (first IVF/ICSI-ET cycle, July 2023–July 2024) were retrospectively analyzed. Patients were stratified into three groups based on the 25th (P25) and 75th (P75) percentiles of the trigger-day LH distribution: Group 1 (< P25, LH < 1.45 IU/L, n=272), Group 2 (P25–P75, 1.45 IU/L ≤ LH ≤ 4.19 IU/L, n=580), and Group 3 (> P75, LH > 4.19 IU/L, n=283). Ovarian response, embryo quality, and pregnancy outcomes were compared; binary logistic regression evaluated LH-pregnancy associations, with subgroup analyses by transfer type, PCOS status, and BMI category.

**Results:**

Group 1 had significantly more follicles ≥14 mm, retrieved/mature/fertilized oocytes, and high-quality embryos than Groups 2 and 3 (all *P <* 0.05), with LH levels negatively correlated with these indicators (all *P <* 0.05). No significant differences in clinical pregnancy or live birth rates after the first embryo transfer were observed among groups (all P > 0.05), consistent across subgroups. After adjusting for confounders including embryo parameters, trigger-day LH grouping was not independently associated with clinical pregnancy rates of the first transfer (Group 2: aOR=0.945, 95%CI 0.649–1.376, P = 0.767; Group 3: aOR=1.130, 95%CI 0.820–1.557, P = 0.457).

**Conclusion:**

Elevated trigger-day LH levels correlate with poorer ovarian response and embryo quality in overweight and obese women on GnRH antagonist protocol, but not with pregnancy outcomes, nor are they an independent predictor of IVF success.

## 1 Introduction

The prevalence of infertility has steadily increased in recent years (1), posing a significant challenge to family planning and the reproductive health of women. Currently, *in vitro* fertilization/intracytoplasmic sperm injection-embryo transfer (IVF/ICSI-ET) serves as one of the most effective therapeutic strategies (2). Among various contributing factors, obesity severely impairs female fertility through multiple mechanisms, including ovulatory dysfunction, oocyte impairment, chronic low-grade inflammation, and compromised endometrial receptivity (3). Clinical studies demonstrate that overweight or obese patients generally exhibit a diminished response to ovarian stimulation. Despite receiving higher doses and prolonged durations of gonadotropins, their trigger-day serum estradiol levels, oocyte yield, high-quality embryo rate, and ultimate live birth rates remain significantly lower compared to normal-weight counterparts (4–6). Notably, overweight status is frequently associated with hypothalamic-pituitary-ovarian (HPO) axis dysfunction, predisposing these individuals to dysregulated luteinizing hormone (LH) secretion rhythms. Elucidating the regulatory mechanism of trigger-day serum LH levels on IVF outcomes under overweight conditions will facilitate the optimization of clinical management protocols.

LH plays a central role in folliculogenesis, oocyte maturation, and the maintenance of endometrial receptivity (7). During IVF cycles, LH must be maintained within a specific “LH window” to provide optimal support for follicular development (8). Suboptimal LH levels impair theca cell function and steroidogenesis, leading to decreased fertilization rates and an elevated risk of embryo aneuploidy (9, 10). Conversely, a premature LH surge during ovarian stimulation triggers premature luteinization, severely compromising oocyte quality and endometrial receptivity (11, 12). Currently, clinical strategies primarily rely on gonadotropin-releasing hormone (GnRH) antagonists to prevent premature LH surges, or exogenous LH supplementation to correct LH deficiency, aiming to stabilize LH concentrations within this optimal window (13).

The correlation between serum LH levels and IVF outcomes exhibits significant population heterogeneity. For instance, higher LH levels under the GnRH antagonist protocol may benefit patients with normal ovarian reserve or polycystic ovary syndrome (PCOS) (14), whereas elevated trigger-day LH predicts adverse pregnancy outcomes in patients with poor ovarian reserve (POR) (15). However, studies investigating the association between trigger-day LH levels and clinical outcomes in overweight patients remain scarce. Recent evidence suggests that obesity alters the metabolic response of granulosa cells to the preovulatory LH surge, thereby increasing the risk of fertilization failure and poor blastocyst development (16). Therefore, this study aimed to explore the specific effects of trigger-day LH levels on embryo quality and pregnancy outcomes in overweight and obese women.

## 2 Materials and methods

### 2.1 Study population

Clinical data of overweight and obese women who underwent IVF/ICSI-ET for assisted reproduction in the Department of Reproduction and Genetics, Affiliated Hospital of Shandong University of Traditional Chinese Medicine, from July 1, 2023, to July 30, 2024, were collected. The study involved a retrospective analysis. The study protocol was reviewed and approved by the Ethics Committee of the Affiliated Hospital of Shandong University of Traditional Chinese Medicine (Approval No. 2025-033-KY). The Ethics Committee of the Affiliated Hospital of Shandong University of Traditional Chinese Medicine evaluated and approved this study protocol and waived the requirement for informed consent due to the study’s retrospective and anonymous design. This study was reported in accordance with the Strengthening the Reporting of Observational Studies in Epidemiology (STROBE) guidelines. The completed STROBE checklist is provided as **Supplementary Table 5**.

#### 2.1.1 Inclusion/exclusion criteria

Inclusion criteria: (1) Age between 22 and 40 years; (2) BMI ≥25 kg/m^2^; (3) Treated with the gonadotropin-releasing hormone antagonist (GnRH-ant) protocol; (4) Complete follow-up data.

Exclusion criteria: (1) Patients with congenital uterine anatomical abnormalities (e.g., uterine septum, unicornuate uterus) or untreated intrauterine space-occupying lesions (e.g., intrauterine adhesion, endometrial polyps); (2) Patients with other reproductive system diseases (e.g., endometriosis, adenomyosis, ovarian cancer); (3) Patients with other systemic diseases that are not suitable for pregnancy (e.g., autoimmune diseases, severe cardiovascular and cerebrovascular diseases); (4) Chromosomal abnormalities in either spouse; (5) Patients with other severe systemic endocrine diseases (e.g., Cushing’s syndrome, congenital adrenal hyperplasia, hyperprolactinemia, uncontrolled thyroid dysfunction, etc.). However, PCOS, as a key overweight-related subtype of interest in this study, was retained and included as a variable for subgroup analysis; (6) Incomplete medical records; (7) Multiple oocyte retrieval cycles or multiple embryo transfer cycles from the same patient. To strictly ensure the statistical independence of observations, only the patient’s first controlled ovarian stimulation (COS) cycle and her very first subsequent embryo transfer event (either a fresh embryo transfer or the first frozen-thawed embryo transfer) were included; (8) Patients who met the freeze-all criteria underwent a frozen-thawed embryo transfer as their first transfer and were included in the frozen-thawed embryo transfer (FET) subgroup. Patients who never underwent any embryo transfer (e.g., no viable embryos) were excluded.

#### 2.1.2 Grouping

All patients received treatment with the GnRH-ant protocol. When the target follicles reached 18 mm in diameter, trigger was performed at 21:00 with 4000–10000 IU of human chorionic gonadotropin (hCG, 2000 IU, Zhuhai Livzon) and/or 0.1–0.2 mg of triptorelin acetate injection (Diphereline^®^, 0.1 mg, Ferring). According to the serum LH levels on the hCG trigger day, patients were stratified into three groups based on the 25th and 75th percentiles (P25, P75) of the study population: Group 1 (LH < 1.45 IU/L, n = 272), Group 2 (1.45 IU/L ≤ LH ≤ 4.19 IU/L, n = 580), and Group 3 (LH > 4.19 IU/L, n = 283). The 25th and 75th percentiles were used to define LH thresholds to ensure balanced group sizes. However, these cutoffs are sample-specific and may not be generalizable to other populations. Binary logistic regression analysis was used to explore the correlation between trigger-day serum LH levels and pregnancy outcomes, and multivariate logistic regression analysis was performed to examine the potential impact of known confounding factors on pregnancy outcomes. Meanwhile, Subgroup analyses were stratified by transfer type, PCOS status, and BMI category based on the first embryo transfer cycle per patient. Of the 1,135 included patients (representing 1,135 first COS cycles), 446 underwent a fresh embryo transfer as their first transfer, and 689 underwent a frozen-thawed embryo transfer as their first transfer (representing a freeze-all strategy). Subsequent embryo transfers from the same oocyte retrieval were excluded to ensure statistical independence. These transfer cycles were stratified by transfer type into the fresh embryo transfer subgroup (A1: LH < 1.45 IU/L, n = 94; A2: 1.45 ≤ LH ≤ 4.19 IU/L, n = 243; A3: LH > 4.19 IU/L, n = 109; total n = 446 cycles) and the frozen-thawed embryo transfer (FET) subgroup (B1: LH < 1.45 IU/L, n = 178; B2: 1.45 ≤ LH ≤ 4.19 IU/L, n = 337; B3: LH > 4.19 IU/L, n = 174; total n = 689 cycles). Stratified by PCOS status into the PCOS subgroup (C1: LH < 1.45 IU/L, n = 61; C2: 1.45 ≤ LH ≤ 4.19 IU/L, n = 162; C3: LH > 4.19 IU/L, n = 102) and the non-PCOS subgroup (D1: LH < 1.45 IU/L, n = 211; D2: 1.45 ≤ LH ≤ 4.19 IU/L, n = 418; D3: LH > 4.19 IU/L, n = 181). According to WHO criteria (17), patients were divided into the overweight group (BMI 25.0–29.9 kg/m^2^) and the obese group (BMI ≥ 30.0 kg/m^2^). Stratified by BMI category into the overweight subgroup (E1: LH < 1.45 IU/L, n = 229; E2: 1.45 ≤ LH ≤ 4.19 IU/L, n = 470; E3: LH > 4.19 IU/L, n = 235) and the obese subgroup (F1: LH < 1.45 IU/L, n = 43; F2: 1.45 ≤ LH ≤ 4.19 IU/L, n = 110; F3: LH > 4.19 IU/L, n = 48).

### 2.2 Treatment methods

Controlled ovarian stimulation was initiated on days 2–3 of the patient’s menstrual cycle with an initial dose of 150–300 U/d of recombinant follicle-stimulating hormone (rFSH) injection (Puregon^®^, 600 IU, Merck Sharp & Dohme; or Gonal-F^®^, Merck-Serono SA, Switzerland) or urinary FSH (Lishenbao; Zhuhai Lizhu Pharmaceutical Co., Ltd.). The dose and type of Gn were determined based on the female’s age, BMI, basal FSH level, and AFC. Serum endocrine levels and follicular development were monitored every 2–4 days. When transvaginal ultrasound revealed that the dominant follicle reached 12–14 mm in diameter, 0.25 mg/d of ganirelix acetate (ORGALUTRAN^®^, 0.25 mg, Merck Sharp & Dohme) was subcutaneously administered until the hCG trigger day. When the target follicle reached 18 mm in diameter, trigger was performed at 21:00 with 4000–10000 IU of human chorionic gonadotropin (hCG, 2000 IU, Zhuhai Livzon Pharmaceutical Group Co., Ltd.) and/or 0.1–0.2 mg of triptorelin acetate injection (Diphereline^®^, 0.1 mg, Ferring Pharmaceuticals). Oocyte retrieval was performed under ultrasound guidance 34–36 hours later, followed by IVF or ICSI (in cases of severe oligozoospermia, asthenospermia, or teratozoospermia in males). Embryo transfer was performed 3 days after embryo culture. All embryos were cryopreserved for elective transfer if any of the following conditions were met: (1) Endometrial thickness ≤ 6 mm or ≥ 15 mm on the hCG trigger day, or presence of endometrial polyps or intrauterine fluid; (2) Progesterone level ≥ 2 ng/mL on the hCG trigger day; (3) Estradiol level ≥ 4800 pg/mL on the hCG trigger day; (4) Number of oocytes retrieved ≥ 15, suggesting a tendency for ovarian hyperstimulation syndrome (OHSS). Progesterone injection was administered for luteal support after embryo transfer. If the serum hCG level was > 25 mIU/mL on day 14 after transfer, luteal support was continued. Clinical pregnancy was confirmed by transvaginal ultrasound performed 28–35 days after transfer, which showed a gestational sac in the uterus.

### 2.3 Outcome measures

The primary outcomes of this study were the clinical pregnancy rate (CPR) and live birth rate (LBR). CPR and LBR were defined as the proportion of patients who achieved a clinical pregnancy or live birth after their first embryo transfer (either fresh or frozen). The analysis included 1,135 patients, with each patient contributing exactly one transfer cycle to ensure statistical independence. The secondary outcomes included the number of oocytes retrieved, number of mature oocytes, and embryological outcomes. Fertilization was assessed 16–18 hours after insemination or ICSI. Total fertilized oocytes were defined as all oocytes showing two pronuclei (2PN) or abnormal fertilization (1PN or ≥3PN). Normal fertilization was specifically defined as the presence of two pronuclei (2PN) and two polar bodies. The total fertilization rate was calculated as the number of total fertilized oocytes divided by the number of MII oocytes, multiplied by 100%. The normal fertilization rate was calculated as the number of 2PN oocytes divided by the number of MII oocytes, multiplied by 100%. Additional outcomes included the number of transferable embryos and the number of high-quality embryos. Additionally, the high-quality embryo rate was defined as the number of high-quality embryos divided by the number of 2PN oocytes. Embryo quality was graded by professional embryologists: cleavage-stage embryos were graded according to the Istanbul Consensus (18), and blastocysts were graded according to the method described by Gardner et al. (19). Cleavage-stage embryos of Grade I and blastocysts of Grade 3BB or higher were considered high-quality embryos. Serum β-hCG was measured on day 14 after embryo transfer; if β-hCG > 25 mIU/mL, luteal support was continued. Transvaginal ultrasound was performed 28–35 days after transfer. Clinical pregnancy was defined as the presence of a gestational sac with a fetal pole and primitive heartbeat detected by ultrasound in the uterus. Live birth was defined as the delivery of a neonate with vital signs at ≥ 28 weeks of gestation; Early miscarriage as termination of pregnancy at < 28 weeks of gestation with a fetal weight < 1000 g. Early miscarriage rate was calculated per clinical pregnancy.

### 2.4 Statistical analysis

SPSS 27.0 statistical software was used for data processing and analysis. Continuous variables with normal distribution were expressed as mean ± standard deviation. One-way analysis of variance (one-way ANOVA) was used for overall comparison among the three groups, and *post-hoc* test based on Bonferroni correction was used for pairwise comparison. Continuous variables with non-normal distribution were expressed as median and interquartile range [M (P25, P75)]. Kruskal-Wallis H test was used for comparison among multiple groups, and Dunn test with Bonferroni correction for P values was used for pairwise comparison. Categorical variables were expressed as percentage and frequency [% (n/N)]. Pearson χ^2^ test or Fisher’s exact test was used for comparison between groups, and P value correction for multiple comparisons between groups was performed when necessary. Spearman rank correlation analysis was used to explore the correlation between trigger-day serum LH levels and various skewed continuous clinical indicators (such as basal endocrine indicators, controlled ovarian stimulation parameters, and embryological indicators). Binary logistic regression analysis was used to evaluate the independent effect of trigger-day serum LH levels on clinical pregnancy outcomes. To strictly control for potential confounders and address intermediate effects, we adopted a stepwise adjustment strategy constructing two models. Model 1 was adjusted for baseline and clinical characteristics, including female age, male age, BMI, PCOS status, trigger modality, fertilization method (IVF vs. ICSI), years of infertility, basal FSH, and AFC. Model 2 (the fully adjusted model) included all covariates in Model 1, with the further inclusion of cycle-specific embryo transfer characteristics: endometrial thickness on the transfer day, transfer of high-quality embryo (Yes vs. No), and total number of transferred embryos. Specifically, categorical variables such as PCOS status were coded as binary variables with the non-PCOS group serving as the reference (1 = PCOS, 0 = non-PCOS). For subgroup analyses, variables used for stratification (e.g., BMI, PCOS status) were appropriately excluded from the covariates of the respective models to prevent collinearity. Furthermore, in subgroups with extremely rare categorical events (e.g., only 1 patient receiving GnRH agonist alone trigger in the fresh ET subgroup), these specific cases were excluded to ensure model stability. The results are expressed as adjusted odds ratios (aORs) with 95% confidence intervals (CIs). A *post-hoc* power calculation was performed to determine the minimum detectable effect size for the primary outcome (clinical pregnancy rate) using a conventional two-sided α of 0.05 and a power of 80% (1-β = 0.80). For the correlation analysis in **Table 1**, which involved 18 clinical and laboratory indicators, the Bonferroni correction was applied to adjust for multiple comparisons. Consequently, the threshold for statistical significance was strictly adjusted to a two-sided *P-*value<0.0028 (0.05/18).To assess the robustness of the primary findings and address potential information loss from data categorization, a sensitivity analysis was performed by analyzing LH strictly as a continuous variable in the multivariable logistic regression model, adjusting for the same confounding factors.

**Table 1:** Correlation analysis of serum LH level on the trigger day.

Variable	r_s_	*P-*value
Female age	0.043	0.146
BMI	0.029	0.330
Infertility duration	0.050	0.091
Basal FSH	0.064	0.031
Basal LH	0.032	0.281
Basal E2	0.015	0.619
Basal P	-0.054	0.068
Basal T	0.023	0.431
AFC	-0.022	0.462
Follicles ≥14 mm on trigger day	-0.156**	<0.001
E2 on trigger day	-0.021	0.474
P on trigger day	-0.028	0.339
Number of oocytes retrieved	-0.163**	<0.001
Number of mature oocytes	-0.173**	<0.001
Number of fertilized oocytes	-0.161**	<0.001
Number of normally fertilized oocytes (2PN)	-0.160**	<0.001
Number of transferable embryos	-0.106**	<0.001
Number of high-quality embryos	-0.131**	<0.001

**P < 0.0028 (Statistically significant after Bonferroni correction for multiple testing).

## 3 Results

### 3.1 Comparison of data among the three groups

**Figure 1:**
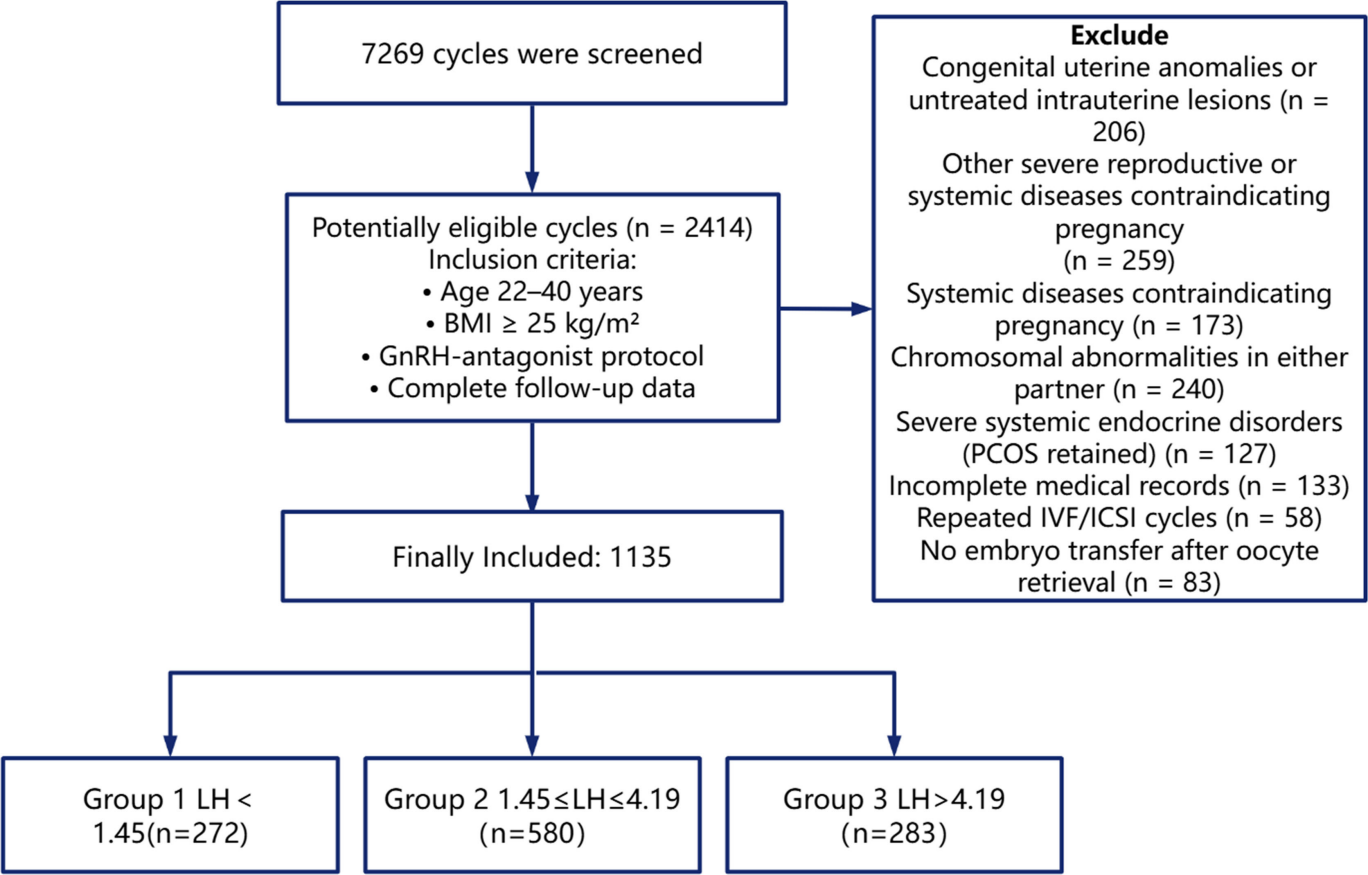
Inclusion and exclusion criteria of the cases.

A total of 7,269 clinical cycles from infertile women were initially screened in this study. To ensure the statistical independence of observations, inclusion was strictly limited to the first controlled ovarian stimulation (COS) cycle of each patient. Ultimately, the clinical data of 1,135 overweight and obese women (representing exactly 1,135 independent first COS cycles) were included in the statistical analysis. The inclusion and exclusion criteria of the cases are shown in **Figure 1**. According to the serum LH level on the hCG trigger day, the subjects were divided into three groups: Group 1 (LH < 1.45 IU/L, n = 272), Group 2 (1.45 IU/L ≤ LH ≤ 4.19 IU/L, n = 580), and Group 3 (LH > 4.19 IU/L, n = 283). There were no statistically significant differences among the three groups in terms of female age, male age, overweight/obesity (including overweight and obese proportions), years of infertility, primary infertility, infertility factors (fallopian tube factors and male factor), basal endocrine indicators (FSH, LH, E2, P, T), AFC, BMI, ovulation trigger protocol (hCG, triptorelin, dual trigger), IVF/ICSI, E2 on trigger day level, P on trigger day level, number of high-quality embryos transferred, endometrial thickness on transfer day, number of embryos transferred, clinical pregnancy rate, and live birth rate (all P > 0.05). Statistically significant differences were observed among the three groups in the proportion of PCOS patients, Gn duration (days), total Gn dosage, number of follicles ≥14 mm on the trigger day, number of retrieved oocytes, number of mature oocytes (MII), number of fertilized oocytes, number of normally fertilized oocytes (2PN), number of available embryos, and number of high-quality embryos (all *P <* 0.05), as detailed in **Tables 2**, **3**. We noted that the median number of high-quality embryos was identical (1 [0–2]) across groups but showed a significant difference by Kruskal-Wallis H test. This discrepancy was due to distinct frequency distributions (0, 1, ≥2 embryos) and rank-based differences rather than median values. Detailed distribution patterns were analyzed using frequency histograms and box plots, as shown in **Supplementary Figure 1**; **Supplementary Table 1**.

**Table 2:** Baseline characteristics of overweight and obese women stratified by LH group.

Group	Group 1 (n=272)	Group 2 (n=580)	Group 3 (n=283)	*P-*value
Female age (years)	32.71 ± 4.21	33.07 ± 4.38	32.91 ± 4.49	0.555
Male age (years)	34.31 ± 5.01	34.33 ± 5.65	34.15 ± 5.75	0.805
Overweight/obese				0.494
Overweight (%)	84.2%(229/272)	81.0%(470/580)	83.0%(235/283)	
Obese (%)	15.8%(43/272)	19.0%(110/580)	17.0%(48/283)	
Duration of infertility (years)	3(2,4)	3(2,5)	3(2,6)	0.126
Primary infertility (%)	44.5%(121/272)	39.3%(228/580)	41.3%(117/283)	0.357
Infertility factors (%)				
Fallopian tube factors (%)	93.4%(254/272)	93.6%(543/580)	95.1%(269/283)	0.649
Male factor (%)	20.6%(56/272)	17.8%(103/580)	18.7%(53/283)	0.614
PCOS (%)	22.4%(61/272)	27.9%(162/580)	36.0%(102/283)**;#	0.002*
Basal FSH (mIU/mL)	6.41(5.34,7.52)	6.45(5.39,7.62)	6.45(5.46,7.85)	0.401
Basal LH (mIU/mL)	5.29 ± 2.44	5.38 ± 2.48	5.69 ± 2.86	0.132
Basal E2 (pg/mL)	37.89 ± 12.05	38.03 ± 11.54	37.89 ± 11.14	0.98
Basal P (ng/mL)	0.41 ± 0.23	0.40 ± 0.25	0.38 ± 0.22	0.274
Basal T (ng/mL)	0.56 ± 0.40	0.53 ± 0.38	0.57 ± 0.41	0.224
AFC (n)	23(17,33)	22(15,31)	22(15,34)	0.23
BMI (kg/m^2^)	28.02 ± 2.45	28.15 ± 2.91	27.98 ± 2.30	0.617
Gn duration (days)	10.13 ± 2.15	9.83 ± 2.48	9.57 ± 1.84**	0.014*
Total Gn dosage (IU)	2604.92 ± 976.15	2472.58 ± 892.51*	2384.72 ± 715.27**	0.012*
Ovulation trigger protocol				0.397
Triggered with hCG	74.6%(203/272)	73.8%(428/580)	70.3%(199/283)	
GnRH agonist alone	1.1%(3/272)	2.8%(16/580)	2.1%(6/283)	
dual trigger	24.3%(66/272)	23.4%(136/580)	27.6%(78/283)	
IVF/ICSI				0.493
IVF	79.8%(217/272)	79.8%(463/580)	83.0%(235/283)	
ICSI	20.2%(55/272)	20.2%(117/580)	17.0%(48/283)	
Number of follicles ≥14 mm on trigger day (n)	12(7.25,19)	10(6,16)**	9(5,16)**	<0.001*
E2 on trigger day (pg/mL)	2579.56 ± 1535.22	2588.71 ± 1643.81	2578.20 ± 1653.95	0.995
P on trigger day (ng/mL)	0.91(0.55,1.27)	0.85(0.55,1.20)	0.84(0.59,1.16)	0.528
Number of retrieved oocytes (n)	12(7,17)	10(5.25,15)**	9(5,14)**	<0.001*
Number of mature oocytes (MII)	10(6,16)	8.50(5,13)**	8(4,12)**	<0.001*
Number of fertilized oocytes (n)	8(5,12)	7(4,11)**	6(3,10)**	<0.001*
Normally fertilized oocytes (2PN)	6(3,9.75)	5(3,9)*	4(2,8)**;#	<0.001*
Total fertilization rate (%, per MII oocyte)	79.66% (2605/3270)	83.19% (4883/5870)**	82.44% (2084/2528)*	<0.001*
Normal fertilization rate (%, per MII oocyte)	61.22% (2002/3270)	64.07% (3761/5870)*	61.87% (1564/2528)	0.015*
Number of available embryos (n)	3.50(2,6)	3(2,5)	3(1,5)**	0.013*
Number of high-quality embryos (n)	1(0,2)	1(0,2)	1(0,2)**;##	0.001*
High-quality embryo rate (%)	23.58% (472/2002)	23.61% (888/3761)	22.89% (358/1564)	0.865
Number of high-quality embryos transferred				0.683
0	36.8%(100/272)	36.2%(210/580)	41.3%(117/283)	
1	59.2%(161/272)	59.7%(346/580)	55.1%(156/283)	
2	4.0%(11/272)	4.1%(24/580)	3.5%(10/283)	
Endometrial thickness on transfer day (mm)	10.25 ± 1.74	10.11 ± 1.99	10.41 ± 1.89	0.089
Number of embryos transferred				0.466
1	33.5%(91/272)	34.7%(201/580)	38.2%(108/283)	
2	66.5%(181/272)	65.3%(379/580)	61.8%(175/283)	

Data are presented as mean ± SD, median (IQR), or n (%). ANOVA, Kruskal–Wallis H test, and chi-square test were used for comparisons.*P < 0.05 vs. Group 1; **P < 0.01 vs. Group 1; #*P <* 0.05 vs. Group 2; ##*P <* 0.01 vs. Group 2. LH, luteinizing hormone; FSH, follicle-stimulating hormone; E2, estradiol; P, progesterone; AFC, antral follicle count; BMI, body mass index; Gn, gonadotropin; ICSI, intracytoplasmic sperm injection; MII, metaphase II; 2PN, two pronuclear. An asterisk (*) in the overall *P-*value column indicates statistical significance (*P <* 0.05) among the three groups.

**Table 3:** Clinical pregnancy outcomes of overweight and obese women stratified by LH group.

Group	Group 1 (n=272)	Group 2 (n=580)	Group 3 (n=283)	*P*-value
Clinical pregnancy rate (%)	43.8%(119/272)	46.9%(272/580)	42.8%(121/283)	0.453
Live birth rate (%)	35.3%(96/272)	35.9%(208/580)	33.2%(94/283)	0.743

Group 1: LH < 1.45 IU/L (below the 25th percentile); Group 2: 1.45 IU/L ≤ LH ≤ 4.19 IU/L (25th–75th percentile); Group 3: LH > 4.19 IU/L (above the 75th percentile). Clinical pregnancy rate (CPR) and Live birth rate (LBR) were calculated based on the outcome of the first embryo transfer (either fresh or frozen-thawed) following the index oocyte retrieval cycle. *P* values were obtained using the chi-square test.

### 3.2 Comparison among subgroups stratified by transfer type

In the subgroup comparison stratified by transfer type (446 fresh transfer cycles and 689 FET cycles derived from the 1,135 initial COS cycles), in the fresh embryo transfer subgroup, no significant differences were observed among the three groups in demographic characteristics (female age, male age), BMI, overweight/obese proportions, infertility characteristics (years of infertility, primary infertility, infertility factors), PCOS, basal endocrine indicators (FSH, LH, E2, P, T), AFC, Gn duration (days), total Gn dosage, IVF/ICSI, E2 on trigger day and P levels, transfer-related parameters (endometrial thickness, number of embryos transferred, number of high-quality embryos transferred), and pregnancy outcomes (clinical pregnancy rate, ectopic gestation rate, early miscarriage rate, live birth rate) (all P > 0.05). However, significant differences were observed in the number of follicles ≥14 mm on the trigger day, number of oocytes retrieved, number of mature oocytes, number of fertilized oocytes, number of normally fertilized oocytes (2PN), number of available embryos, and number of high-quality embryos (all *P <* 0.05), as detailed in **Table 4**.

**Table 4:** Baseline characteristics of fresh embryo transfer cycles.

Group	A1 (n=94)	A2 (n=243)	A3 (n=109)	*P*-value
Female age (years)	33.54 ± 4.25	33.40 ± 4.40	33.63 ± 4.55	0.887
Male age (years)	34.70 ± 5.74	34.73 ± 5.75	35.13 ± 6.25	0.822
Overweight/obese				0.403
Overweight (%)	85.1% (80/94)	79.8% (194/243)	84.4% (92/109)	
Obese (%)	14.9% (14/94)	20.2% (49/243)	15.6% (17/109)	
Duration of infertility (years)	3 (2,5)	3 (2,5)	3 (2,6)	0.503
Primary infertility (%)	42.6% (40/94)	35.0% (85/243)	36.7% (40/109)	0.433
Infertility factors (%)				
Fallopian tube factors	93.6% (88/94)	93.0% (226/243)	94.5% (103/109)	0.87
Male factor	19.1% (18/94)	18.9% (46/243)	21.1% (23/109)	0.889
PCOS (%)	9.6% (9/94)	17.3% (42/243)	15.6% (17/109)	0.209
Basal FSH (mIU/mL)	6.98 (5.65,8.15)	6.52 (5.49,7.77)	7.02 (5.94,8.51)	0.54
Basal LH (mIU/mL)	4.89 ± 1.83	5.18 ± 2.31	4.76 ± 2.21	0.205
Basal E2 (pg/mL)	37.58 ± 11.04	37.80 ± 11.44	37.24 ± 11.29	0.91
Basal P (ng/mL)	0.41 ± 0.23	0.38 ± 0.25	0.40 ± 0.23	0.514
Basal T (ng/mL)	0.49 ± 0.31	0.49 ± 0.36	0.51 ± 0.35	0.899
AFC (n)	20 (13,25)	20 (13,26)	20 (13,25)	0.798
BMI (kg/m2)	27.75 ± 2.18	28.18 ± 3.24	28.04 ± 2.46	0.459
Gn duration (days)	9.85 ± 1.91	9.65 ± 2.28	9.22 ± 1.70	0.076
Total Gn dosage (IU)	2569.55 ± 956.17	2494.96 ± 872.30	2350.69 ± 650.74	0.159
IVF/ICSI				0.222
IVF	87.2% (82/94)	79.4% (193/243)	83.5% (91/109)	
ICSI	12.8% (12/94)	20.6% (50/243)	16.5% (18/109)	
Number of follicles ≥14 mm on trigger day	9 (6,12)	8 (5,11)	7 (4,10)**	0.004*
E2 on trigger day (pg/mL)	1814.96 (1587.45, 2042.47)	1962.68 (1817.78, 2107.58)	1758.58 (1568.20, 1948.97)	0.225
P on trigger day (ng/mL)	0.67 (0.42,1.02)	0.79 (0.49,1.02)	0.8 (0.55,1.16)	0.136
Number of retrieved oocytes	9 (5,12)	8 (5,11)	7 (4,10)**	0.011*
Number of mature oocytes (MII)	8 (5,11)	7 (4,10)	6 (3.5,9)**;#	0.012*
Number of fertilized oocytes	8 (4,10)	7 (4,9)	5 (3,8)**;##	<0.001*
Normally fertilized oocytes (2PN)	6 (3,7.25)	5 (3,7)	4 (2,5.5)**;##	0.003*
Total fertilization rate (%, per MII oocyte)	85.27% (608/713)	84.51% (1440/1704)	87.04% (591/679)	0.28
Normal fertilization rate (%, per MII oocyte)	65.64% (468/713)	67.55% (1151/1704)	65.83% (447/679)	0.54
Number of available embryos (n)	2 (2,4)	3 (2,4)	2 (1,4)	0.086
Number of high-quality embryos (n)	1 (1,2)	1 (0,2)	1 (0,1)**;##	<0.001*
High-quality embryo rate (%)	27.99% (131/468)	27.11% (312/1151)	20.81% (93/447)*;#	0.019*
Number of high-quality embryos transferred				0.108
0	35.1%(33/94)	34.6%(84/243)	48.6%(53/109)	
1	58.5%(55/94)	60.1%(146/243)	45.0%(49/109)	
2	6.4%(6/94)	5.3%(13/243)	6.4%(7/109)	
Endometrial thickness on transfer day (mm)	10.71 ± 1.79	10.42 ± 2.02	10.82 ± 1.95	0.152
Number of embryos transferred				0.376
1	19.1%(18/94)	23.9%(58/243)	27.5%(30/109)	
2	80.9%(76/94)	76.1%(185/243)	72.5%(79/109)	
Clinical pregnancy rate (%)	40.4%(38/94)	46.1%(112/243)	44.0%(48/109)	0.641
Ectopic gestation rate (%)	2.6%(1/38)	0.9%(1/112)	2.1%(1/48)	0.701
Early miscarriage rate (%)	15.8%(6/38)	22.3%(25/112)	16.7%(8/48)	0.567
Live birth rate (%)	33.0%(31/94)	30.5%(74/243)	33.0%(36/109)	0.846

A1, LH < 1.45 IU/L; A2, 1.45 IU/L ≤ LH ≤ 4.19 IU/L; A3, LH > 4.19 IU/L. Normally distributed continuous data are presented as mean ± SD, and non-normally distributed data as median (P25, P75). *P < 0.05 vs. A1; **P < 0.01 vs. A1; #*P <* 0.05 vs. A2; ##*P <* 0.01 vs. A2. Clinical pregnancy and live birth rates are calculated per embryo transfer cycle. Rates for ectopic gestation and early miscarriage were calculated per clinical pregnancy. An asterisk (*) in the overall *P-*value column indicates statistical significance (*P <* 0.05) among the three groups.

In the FET subgroup, no significant differences were observed among the three groups in demographic characteristics (female age, male age), BMI, overweight/obese proportions, infertility characteristics (years of infertility, primary infertility, infertility factors), basal endocrine indicators (FSH, LH, E2, P, T), AFC, Gn duration (days), total Gn dosage, IVF/ICSI, E2 on trigger day and P levels, transfer-related parameters (endometrial thickness, number of embryos transferred, number of high-quality embryos transferred), and pregnancy outcomes (clinical pregnancy rate, ectopic gestation rate, early miscarriage rate, live birth rate) (all P > 0.05). However, significant differences were observed in the proportion of PCOS, number of follicles ≥14 mm on the trigger day, number of oocytes retrieved, number of mature oocytes, number of fertilized oocytes, and Number of normally fertilized oocytes (2PN) (all *P <* 0.05), as detailed in **Table 5**.

**Table 5:** Baseline characteristics of frozen-thawed embryo transfer cycles.

Group	B1 (n=178)	B2 (n=337)	B3 (n=174)	*P*-value
Female age (years)	32.26 ± 4.13	32.83 ± 4.36	32.45 ± 4.40	0.326
Male age (years)	34.10 ± 4.58	34.04 ± 5.57	33.53 ± 5.34	0.519
Overweight/obese				0.872
Overweight (%)	83.7%(149/178)	81.9%(276/337)	82.2%(143/174)	
Obese (%)	16.3%(29/178)	18.1%(61/337)	17.8%(31/174)	
Duration of infertility (years)	3(2,4)	3(2,6)	3(2,5.25)	0.192
Primary infertility (%)	45.5%(81/178)	42.4%(143/337)	44.3%(77/174)	0.788
Infertility factors (%)				
Fallopian tube factors	93.3%(166/178)	94.1%(317/337)	95.4%(166/174)	0.684
Male factor	21.3%(38/178)	16.9%(57/337)	17.2%(30/174)	0.434
PCOS (%)	29.2%(52/178)	35.6%(120/337)	48.9%(85/174)**;#	<0.001*
Basal FSH (mIU/mL)	6.14(5.31,7.19)	6.39(5.33,7.5)	6.11(5.26,7.51)	0.304
Basal LH (mIU/mL)	5.50 ± 2.68	5.52 ± 2.60	6.28 ± 3.06**;##	0.006*
Basal E2 (pg/mL)	38.05 ± 12.58	38.19 ± 11.63	38.31 ± 11.06	0.979
Basal P (ng/mL)	0.41 ± 0.23	0.41 ± 0.24	0.37 ± 0.21	0.134
Basal T (ng/mL)	0.60 ± 0.44	0.55 ± 0.38	0.61 ± 0.44	0.232
AFC (n)	26(18,35.25)	23(17,35)	25(17,36)	0.006
BMI (kg/m2)	28.16 ± 2.58	28.13 ± 2.64	27.95 ± 2.20	0.664
Gn duration (days)	10.28 ± 2.25	9.96 ± 2.62	9.78 ± 1.90	0.135
Total Gn dosage (IU)	2623.60 ± 988.70	2456.44 ± 907.74	2406.03 ± 753.92	0.051
IVF/ICSI				0.262
IVF	75.8%(135/178)	80.1%(270/337)	82.8%(144/174)	
ICSI	24.2%(43/178)	19.9%(67/337)	17.2%(30/174)	
Number of follicles ≥14 mm on trigger day	16(10,21)	14(7,20)**	13(7,19)**	0.005*
E2 on trigger day (pg/mL)	2983.33 ± 1576.66	3040.12 ± 1794.84	3091.64 ± 1772.83	0.842
P on trigger day (ng/mL)	1.04(0.68,1.49)	0.94(0.58,1.41)	0.87(0.6,1.19)	0.06
Number of retrieved oocytes (n)	16(9,21)	13(6,19)*	12(6,17)**	0.001*
Number of mature oocytes (MII)	13(7,19)	11(5,17)**	9.5(5,15)**	<0.001*
Number of fertilized oocytes (n)	11(6,17)	10(4,15)*	9(4,15)**	0.011*
Normally fertilized oocytes (2PN)	9(4.75,13)	7(3,12)*	6(3,11.25)**	0.003*
Total fertilization rate (%, per MII oocyte)	78.06%(1993/2553)	82.65%(3443/4166)**	80.75%(1493/1849)*	<0.001*
Normal fertilization rate (%, per MII oocyte)	59.93%(1530/2553)	62.65%(2610/4166)	60.41%(1117/1849)	0.054
Number of available embryos (n)	4(2,6)	4(2,6)	3(2,6)	0.09
Number of high-quality embryos (n)	2(0,3)	1(0,3)	1(0,2)	0.132
High-quality embryo rate (%)	22.29%(341/1530)	22.07%(576/2610)	23.72%(265/1117)	0.53
Number of high-quality embryos transferred				0.894
0	37.6%(67/178)	37.4%(126/337)	36.8%(64/174)	
1	59.6%(106/178)	59.3%(200/337)	61.5%(107/174)	
2	2.8%(5/178)	3.3%(11/337)	1.7%(3/174)	
Endometrial thickness on transfer day (mm)	10.01 ± 1.67	9.89 ± 1.94	10.22 ± 1.86	0.166
Number of embryos transferred				0.764
1	41.0%(73/178)	42.4%(143/337)	44.8%(78/174)	
2	59.0%(105/178)	57.6%(194/337)	55.2%(96/174)	
Clinical pregnancy rate (%)	45.5%(81/178)	47.5%(160/337)	42.0%(73/174)	0.494
Ectopic gestation rate (%)	2.5%(2/81)	2.5%(4/160)	4.1%(3/73)	0.768
Early miscarriage rate (%)	12.3%(10/81)	10.0%(16/160)	11.0%(8/73)	0.857
Live birth rate (%)	36.5%(65/178)	39.8%(134/337)	33.3%(58/174)	0.351

B1, LH < 1.45 IU/L; B2, 1.45 IU/L ≤ LH ≤ 4.19 IU/L; B3, LH > 4.19 IU/L. Normally distributed continuous data are presented as mean ± SD, and non-normally distributed data as median (P25, P75). *P < 0.05 compared with B1; **P < 0.01 compared with B1; #*P <* 0.05 compared with B2; ##*P <* 0.01 compared with B2.Clinical pregnancy and live birth rates are calculated per embryo transfer cycle. Rates for ectopic gestation and early miscarriage were calculated per clinical pregnancy. An asterisk (*) in the overall *P-*value column indicates statistical significance (*P <* 0.05) among the three groups.

### 3.3 Comparison among subgroups stratified by PCOS status

In the PCOS subgroup, there were no significant differences among the three groups in terms of demographic characteristics, baseline endocrine indicators, transfer-related parameters, and pregnancy outcomes (all P > 0.05); significant differences were observed in the number of follicles ≥14 mm on the trigger day, number of oocytes retrieved, number of mature oocytes, number of fertilized oocytes, number of normally fertilized oocytes (2PN), and number of transferable embryos (all *P <* 0.05), as shown in **Table 6**.

**Table 6:** Baseline characteristics of PCOS patients in each LH group.

Group	C1 (n=61)	C2 (n=162)	C3 (n=102)	*P*-value
Female age (years)	31.30 ± 4.36	30.56 ± 4.09	30.38 ± 3.90	0.362
Male age (years)	32.87 ± 4.25	31.86 ± 4.28	31.41 ± 4.34	0.111
Overweight/obese				0.782
Overweight (%)	80.3%(49/61)	75.9%(123/162)	77.5%(79/102)	
Obese (%)	19.7%(12/61)	24.1%(39/162)	22.5%(23/102)	
Duration of infertility (years)	3(2,5)	4(2,6)	3(2,6)	0.499
Primary infertility (%)	50.8%(31/61)	54.3%(88/162)	56.9%(58/102)	0.754
Fallopian tube factors	95.1%(58/61)	93.8%(152/162)	95.1%(97/102)	0.883
Male factor	16.4%(10/61)	12.3%(20/162)	16.7%(17/102)	0.557
Basal FSH (mIU/mL)	6.00(5.04,6.62)	6.10(5.14,6.88)	5.84(5.09,6.93)	0.842
Basal LH (mIU/mL)	7.18 ± 3.21	7.08 ± 3.20	7.92 ± 3.09	0.1
Basal E2 (pg/mL)	38.59 ± 11.39	38.31 ± 12.30	38.26 ± 11.09	0.983
Basal P (ng/mL)	0.38 ± 0.20	0.42 ± 0.26	0.38 ± 0.21	0.405
Basal T (ng/mL)	0.68 ± 0.35	0.64 ± 0.37	0.68 ± 0.42	0.61
AFC (n)	36(30,42)	31(23,42.25)	34(24,45.25)	0.115
BMI (kg/m2)	28.52 ± 2.56	28.46 ± 2.76	28.35 ± 2.49	0.907
Gn duration (days)	10.46 ± 2.60	10.43 ± 3.23	9.87 ± 1.80	0.224
Total Gn dosage (IU)	2559.43 ± 1063.46	2389.69 ± 974.12	2261.89 ± 685.12	0.131
IVF/ICSI				0.431
IVF	88.5%(54/61)	83.3%(135/162)	88.2%(90/102)	
ICSI	11.5%(7/61)	16.7%(27/162)	11.8%(12/102)	
Number of follicles ≥14 mm on trigger day	21(16,27)	17(11.75,23)**	17(11,22.25)**	0.006*
E2 on trigger day (pg/mL)	3588.37 ± 1535.43	3683.12 ± 1679.44	3655.70 ± 1637.18	0.929
P on trigger day (ng/mL)	1.10(0.71,1.65)	1.01(0.63,1.40)	0.90(0.62,1.33)	0.239
Number of retrieved oocytes (n)	18(12.5,27.5)	16.5(10,22)	14(10,21)*	0.018*
Number of mature oocytes (MII)	17(11,24)	14(8.75,20)	12(8,18)**	0.003*
Number of fertilized oocytes (n)	13(8.5,20)	12(7,16.25)	9(6,15)**	0.003*
Normally fertilized oocytes (2PN)	11(7,15)	8(4,14)	6.5(3,12)**	0.001*
Total fertilization rate (%, per MII oocyte)	79.75% (906/1136)	83.44% (2096/2512)*	79.94% (1092/1366)#	0.005*
Normal fertilization rate (%, per MII oocyte)	62.85% (714/1136)	62.38% (1567/2512)	59.30% (810/1366)	0.108
Number of available embryos (n)	6(3,9.5)	4(3,7)*	4(2,7)**	0.022*
Number of high-quality embryos (n)	2(1,4)	2(1,3)	2(0,3)	0.086
High-quality embryo rate (%)	23.39% (167/714)	22.02% (345/1567)	24.07% (195/810)	0.446
Number of high-quality embryos transferred				0.887
0	23.0%(14/61)	26.5%(43/162)	30.4%(31/102)	
1	72.1%(44/61)	69.1%(112/162)	65.7%(67/102)	
2	4.9%(3/61)	4.3%(7/162)	3.9%(4/102)	
Endometrial thickness on transfer day (mm)	10.30 ± 1.53	9.93 ± 1.92	10.13 ± 1.81	0.36
Number of embryos transferred				0.706
1	24.6%(15/61)	30.2%(49/162)	28.4%(29/102)	
2	75.4%(46/61)	69.8%(113/162)	71.6%(73/102)	
Clinical pregnancy rate (%)	52.5%(32/61)	55.6%(90/162)	55.9%(57/102)	0.9
Live birth rate (%)	39.3%(24/61)	39.5%(64/162)	44.1%(45/102)	0.731
Ectopic gestation rate (%)	0.0%(0/32)	3.3%(3/90)	5.3%(3/57)	0.416
Early miscarriage rate (%)	18.8%(6/32)	16.7%(15/90)	12.3%(7/57)	0.672

C1, LH < 1.45 IU/L; C2, 1.45 IU/L ≤ LH ≤ 4.19 IU/L; C3, LH > 4.19 IU/L. Normally distributed continuous data are presented as mean ± SD, and non-normally distributed data as median (P25, P75). *P < 0.05 compared with C1; **P < 0.01 compared with C1; #*P <* 0.05 compared with C2; ##*P <* 0.01 compared with C2.Clinical pregnancy and live birth rates are calculated per embryo transfer cycle. Rates for ectopic gestation and early miscarriage were calculated per clinical pregnancy. An asterisk (*) in the overall *P-*value column indicates statistical significance (*P <* 0.05) among the three groups. All patients included in this table completed exactly one embryo transfer cycle (either fresh or frozen). Patients who never underwent any embryo transfer were excluded.

In the non-PCOS subgroup, there were no significant differences among the three groups in terms of demographic characteristics, baseline endocrine indicators (except FSH), transfer-related parameters (except endometrial thickness), and pregnancy outcomes (all P > 0.05); significant differences were observed in female ages, years of infertility, basal FSH, Gn administration days, endometrial thickness on transfer day, number of follicles ≥14 mm on the trigger day, number of retrieved oocytes, number of mature oocytes (MII), number of fertilized oocytes, number of normally fertilized oocytes (2PN), number of available embryos, and number of high-quality embryos (all *P <* 0.05), as shown in **Table 7**.

**Table 7:** Baseline characteristics of non-PCOS patients in each LH group.

Group	D1 (n=211)	D2 (n=418)	D3 (n=181)	*P*-value
Female age (years)	33.11 ± 4.09	34.04 ± 4.09**	34.33 ± 4.17**	0.007*
Male age (years)	34.72 ± 5.14	35.29 ± 5.83	35.69 ± 5.87	0.23
Overweight/obese				0.556
Overweight (%)	85.3%(180/211)	83.0%(347/418)	86.2%(156/181)	
Obese (%)	14.7%(31/211)	17.0%(71/418)	13.8%(25/181)	
Duration of infertility (years)	3(2,4)	3(2,5)	3(2,5.5)	0.354
Primary infertility (%)	42.7%(90/211)	33.5%(140/418)*	32.6%(59/181)*	0.048*
Fallopian tube factors	92.9%(196/211)	93.5%(391/418)	95.0%(172/181)	0.673
Male factor	21.8%(46/211)	19.9%(83/418)	19.9%(36/181)	0.835
Basal FSH (mIU/mL)	6.60(5.52,7.65)	6.61(5.59,7.90)	6.90(5.79,8.63)	0.033*
Basal LH (mIU/mL)	4.74 ± 1.84	4.72 ± 1.75	4.44 ± 1.75	0.162
Basal E2 (pg/mL)	37.68 ± 12.25	37.92 ± 11.25	37.69 ± 11.19	0.959
Basal P (ng/mL)	0.42 ± 0.23	0.40 ± 0.24	0.38 ± 0.22	0.199
Basal T (ng/mL)	0.53 ± 0.41	0.49 ± 0.37	0.51 ± 0.39	0.373
AFC (n)	21(15,28)	20(13,26)	20(13,25)	0.054
BMI (kg/m2)	27.88 ± 2.41	28.03 ± 2.96	27.78 ± 2.17	0.519
Gn duration (days)	10.03 ± 2.00	9.60 ± 2.08*	9.39 ± 1.85**	0.004*
Total Gn dosage (IU)	2618.07 ± 951.71	2504.70 ± 857.88	2453.94 ± 724.41	0.138
IVF/ICSI				0.789
IVF	77.3%(163/211)	78.5%(328/418)	80.1%(145/181)	
ICSI	22.7%(48/211)	21.5%(90/418)	19.9%(36/181)	
Number of follicles ≥14 mm on trigger day	11(7,16)	8(5,13)**	7(4,10.5)**;##	<0.001*
E2 on trigger day (pg/mL)	2287.91 ± 1409.56	2164.56 ± 1420.29	1971.00 ± 1324.03	0.08
P on trigger day (ng/mL)	0.87(0.54,1.20)	0.81(0.53,1.12)	0.82(0.54,1.13)	0.694
Number of retrieved oocytes (n)	10(6,15)	8(5,12)**	6(3.5,10)**;##	<0.001*
Number of mature oocytes (MII)	9(6,13)	7(4,11)**	5(3,8.5)**;##	<0.001*
Number of fertilized oocytes (n)	7(4,11)	6(3,9)**	5(2,8)**;##	<0.001*
Normally fertilized oocytes (2PN)	5(3,8)	4(2,7)*	3(1,5.5)**;##	<0.001*
Total fertilization rate (%, per MII oocyte)	79.62% (1699/2134)	83.00% (2787/3358)**	85.37% (992/1162)**	<0.001*
Normal fertilization rate (%, per MII oocyte)	60.36% (1288/2134)	65.34% (2194/3358)**	64.89% (754/1162)*	<0.001*
Number of available embryos (n)	3(2,4)	3(2,4)	2(1,4)**;##	<0.001*
Number of high-quality embryos (n)	1(0,2)	1(0,2)	1(0,1)**;##	<0.001*
High-quality embryo rate (%)	23.68% (305/1288)	24.75% (543/2194)	21.62% (163/754)	0.17
Number of high-quality embryos transferred				0.535
0	40.8%(86/211)	40.0%(167/418)	47.5%(86/181)	
1	55.5%(117/211)	56.0%(234/418)	49.2%(89/181)	
2	3.8%(8/211)	4.1%(17/418)	3.3%(6/181)	
Endometrial thickness on transfer day (mm)	10.24 ± 1.80	10.18 ± 2.02	10.63 ± 1.95*;##	0.029*
Number of embryos transferred				0.194
1	36.0%(76/211)	36.4%(152/418)	43.6%(79/181)	
2	64.0%(135/211)	63.6%(266/418)	56.4%(102/181)	
Clinical pregnancy rate (%)	41.2%(87/211)	43.5%(182/418)	35.4%(64/181)	0.174
Live birth rate (%)	34.1%(72/211)	34.4%(144/418)	27.1%(49/181)	0.185
Ectopic gestation rate (%)	3.4%(3/87)	1.1%(2/182)	1.6%(1/64)	0.394
Early miscarriage rate (%)	11.5%(10/87)	14.3%(26/182)	14.1%(9/64)	0.813

D1, LH < 1.45 IU/L; D2, 1.45 IU/L ≤ LH ≤ 4.19 IU/L; D3, LH > 4.19 IU/L. Normally distributed continuous data are presented as mean ± SD, and non-normally distributed data as median (P25, P75). *P < 0.05 compared with D1; **P < 0.01 compared with D1; #*P <* 0.05 compared with D2; ##*P <* 0.01 compared with D2. Clinical pregnancy and live birth rates are calculated per embryo transfer cycle. Rates for ectopic gestation and early miscarriage were calculated per clinical pregnancy. An asterisk (*) in the overall *P-*value column indicates statistical significance (*P <* 0.05) among the three groups.

### 3.4 Comparison among subgroups stratified by BMI category

In the overweight subgroup, there were no significant differences among the three groups in terms of demographic characteristics, baseline endocrine indicators, transfer-related parameters, and pregnancy outcomes (all P > 0.05); significant differences were observed in the proportion of PCOS, Gn administration days, number of follicles ≥14 mm on the trigger day, number of oocytes retrieved, number of mature oocytes, number of fertilized oocytes, number of normally fertilized oocytes (2PN), number of available embryos, and number of high-quality embryos (all *P <* 0.05), as shown in **Table 8**.

**Table 8:** Baseline characteristics of overweight patients (BMI 25.0-29.9 kg/m^2^) in each LH group.

Group	E1 (n=229)	E2 (n=470)	E3 (n=235)	*P*-value
Female age (years)	32.66 ± 4.16	33.21 ± 4.26	33.13 ± 4.46	0.269
Male age (years)	34.12 ± 5.07	34.35 ± 5.55	34.42 ± 5.86	0.828
Duration of infertility (years)	3 (2, 4)	3 (2, 5)	3 (2, 5)	0.172
Primary infertility (%)	44.1%(101/229)	38.7%(182/470)	39.1%(92/235)	0.37
Fallopian tube factors	94.3%(216/229)	93.2%(438/470)	94.9%(223/235)	0.641
Male factor	19.7%(45/229)	17.4%(82/470)	17.0%(40/235)	0.716
PCOS (%)	21.4%(49/229)	26.2%(123/470)	33.6%(79/235)**;#	0.011*
Basal FSH (mIU/mL)	6.40 (5.35, 7.51)	6.51 (5.49, 7.69)	6.57 (5.50, 7.94)	0.222
Basal LH (mIU/mL)	5.20 ± 2.31	5.35 ± 2.48	5.61 ± 2.85	0.199
Basal E2 (pg/mL)	38.30 ± 12.36	37.84 ± 11.49	37.60 ± 11.03	0.801
Basal P (ng/mL)	0.42 ± 0.23	0.40 ± 0.24	0.38 ± 0.22	0.325
Basal T (ng/mL)	0.57 ± 0.41	0.51 ± 0.35	0.56 ± 0.41	0.129
AFC (n)	23 (18, 33)	22 (15.75, 31)	22 (15, 32)	0.266
BMI (kg/m2)	27.17 ± 1.34	27.10 ± 1.25	27.15 ± 1.25	0.788
Gn duration (days)	9.92 ± 1.96	9.71 ± 2.42	9.39 ± 1.81**	0.031*
Total Gn dosage (IU)	2487.88 ± 844.09	2416.29 ± 878.38	2314.15 ± 702.94	0.075
IVF/ICSI				0.6
IVF	79.9%(183/229)	80.0%(376/470)	83.0%(195/235)	
ICSI	20.1%(46/229)	20.0%(94/470)	17.0%(40/235)	
Number of follicles ≥14 mm on trigger day	13 (8, 20)	10 (6, 16)**	9 (5, 15)**	<0.001*
E2 on trigger day (pg/mL)	2631.39 ± 1515.00	2601.51 ± 1644.52	2599.53 ± 1696.67	0.97
P on trigger day (ng/mL)	0.93 (0.58, 1.32)	0.84 (0.55, 1.23)	0.86 (0.58, 1.20)	0.295
Number of retrieved oocytes (n)	12 (7, 17.5)	10 (5.75, 15)**	8 (5, 14)**	<0.001*
Number of mature oocytes (MII)	11 (6.5, 16.5)	9 (5, 14)**	8 (4, 12)**;#	<0.001*
Number of fertilized oocytes (n)	8 (5, 13)	7 (4, 11)**	6 (3, 10)**;#	<0.001*
Normally fertilized oocytes (2PN)	7 (3, 10)	5 (3, 9)**	4 (2, 8)**;#	<0.001*
Total fertilization rate (%, per MII oocyte)	80.15% (2285/2851)	83.24% (3987/4790)**	81.58% (1710/2096)	<0.001*
Normal fertilization rate (%, per MII oocyte)	61.66% (1758/2851)	64.36% (3083/4790)	61.88% (1297/2096)#	0.028*
Number of available embryos (n)	4 (2, 6)	3 (2, 6)	3 (1, 5)**;#	0.005*
Number of high-quality embryos (n)	1 (0.5, 3)	1 (0, 2)	1 (0, 2)**;#	<0.001*
High-quality embryo rate (%)	24.29% (427/1758)	24.68% (761/3083)	23.36% (303/1297)	0.638
Number of high-quality embryos transferred				0.522
0	34.1%(78/229)	33.8%(159/470)	40.0%(94/235)	
1	61.6%(141/229)	62.3%(293/470)	57.0%(134/235)	
2	4.4%(10/229)	3.8%(18/470)	3.0%(7/235)	
Endometrial thickness on transfer day (mm)	10.21 ± 1.77	10.18 ± 2.01	10.46 ± 1.84	0.157
Number of embryos transferred				0.383
1	33.2%(76/229)	35.1%(165/470)	39.1%(92/235)	
2	66.8%(153/229)	64.9%(305/470)	60.9%(143/235)	
Clinical pregnancy rate (%)	47.6%(109/229)	48.3%(227/470)	45.5%(107/235)	0.785
Live birth rate (%)	38.9%(89/229)	37.9%(178/470)	35.3%(83/235)	0.709
Ectopic gestation rate (%)	2.8%(3/109)	1.8%(4/227)	3.7%(4/107)	0.544
Early miscarriage rate (%)	11.9%(13/109)	12.3%(28/227)	14.0%(15/107)	0.881

E1, LH < 1.45 IU/L; E2, 1.45 IU/L ≤ LH ≤ 4.19 IU/L; E3, LH > 4.19 IU/L. Normally distributed continuous data are presented as mean ± SD, and non-normally distributed data as median (P25, P75). *P < 0.05 compared with E1; **P < 0.01 compared with E1; #*P <* 0.05 compared with E2; ##*P <* 0.01 compared with E2. Clinical pregnancy and live birth rates are calculated per embryo transfer cycle. Rates for ectopic gestation and early miscarriage were calculated per clinical pregnancy. An asterisk (*) in the overall *P-*value column indicates statistical significance (*P <* 0.05) among the three groups. According to the World Health Organization (WHO) criteria, this table specifically presents data for the overweight subgroup (BMI 25.0–29.9 kg/m^2^).

In the obese subgroup, there were no significant differences among the three groups in terms of demographic characteristics, baseline endocrine indicators, transfer-related parameters, and pregnancy outcomes (all P > 0.05); a significant difference was observed only in total Gn dosage (*P <* 0.05), as shown in **Table 9**.

**Table 9:** Baseline characteristics of obese patients (BMI ≥ 30.0 kg/m^2^) in each LH group.

Group	F1 (n=43)	F2 (n=110)	F3 (n=48)	*P*-value
Female age (years)	32.95 ± 4.51	32.46 ± 4.82	31.81 ± 4.50	0.503
Male age (years)	35.28 ± 4.61	34.26 ± 6.09	32.83 ± 5.02	0.107
Duration of infertility (years)	3(2,5)	3(1,6)	3(2,6)	0.67
Primary infertility (%)	46.5%(20/43)	41.8%(46/110)	52.1%(25/48)	0.483
Fallopian tube factors	88.4%(38/43)	95.5%(105/110)	95.8%(46/48)	0.209
Male factor	25.6%(11/43)	19.1%(21/110)	27.1%(13/48)	0.461
PCOS (%)	27.9%(12/43)	35.5%(39/110)	47.9%(23/48)	0.129
Basal FSH (mIU/mL)	6.42(5.30,7.55)	6.20(5.16,7.24)	5.98(5.25,7.30)	0.857
Basal LH (mIU/mL)	5.76 ± 3.00	5.49 ± 2.50	6.08 ± 2.90	0.448
Basal E2 (pg/mL)	35.68 ± 10.06	38.86 ± 11.80	39.33 ± 11.70	0.236
Basal P (ng/mL)	0.41 ± 0.23	0.39 ± 0.25	0.38 ± 0.22	0.843
Basal T (ng/mL)	0.54 ± 0.35	0.59 ± 0.46	0.64 ± 0.42	0.509
AFC (n)	22(17,32)	21.50(13,31.25)	27(20.25,37.75)	0.055
BMI (kg/m2)	32.54 ± 2.03	32.64 ± 3.62	32.08 ± 1.83	0.544
Gn duration (days)	11.26 ± 2.70	10.35 ± 2.69	10.42 ± 1.80	0.122
Total Gn dosage (IU)	3228.20 ± 1344.65	2713.07 ± 916.23**	2730.21 ± 680.23*	0.011*
IVF/ICSI				0.813
IVF	79.1%(34/43)	79.1%(87/110)	83.3%(40/48)	
ICSI	20.9%(9/43)	20.9%(23/110)	16.7%(8/48)	
Number of follicles ≥14 mm on trigger day	12(5,16)	10(5.75,16)	10(6,17)	0.965
E2 on trigger day (pg/mL)	2303.53 ± 1629.46	2534.05 ± 1647.13	2473.81 ± 1437.96	0.725
P on trigger day (ng/mL)	0.76(0.37,1.13)	0.87(0.54,1.15)	0.76(0.60,1.02)	0.336
Number of retrieved oocytes (n)	9(5,15)	10(5,14)	10(5,14)	0.961
Mature oocytes (MII)	8(5,13)	8(4.75,13)	7(4.25,12)	0.908
Number of fertilized oocytes (n)	6(4,9)	6(3,11.5)	6.50(4,10.75)	0.985
Normally fertilized oocytes (2PN)	5(3,7)	5(2.75,8.25)	5(3,7)	0.814
Total fertilization rate (%, per MII oocyte)	76.37% (320/419)	82.96% (896/1080)*	86.57% (374/432)**	<0.001*
Normal fertilization rate (%, per MII oocyte)	58.23% (244/419)	62.78% (678/1080)	61.81% (267/432)	0.224
Number of available embryos (n)	2(2,4)	3(2,4)	2(2,4.75)	0.736
Number of high-quality embryos (n)	1(0,1)	1(0,2)	1(0,2)	0.823
High-quality embryo rate (%)	18.44% (45/244)	18.73% (127/678)	20.60% (55/267)	0.76
Number of high-quality embryos transferred				0.908
0	51.2%(22/43)	46.4%(51/110)	47.9%(23/48)	
1	46.5%(20/43)	48.2%(53/110)	45.8%(22/48)	
2	2.3%(1/43)	5.5%(6/110)	6.3%(3/48)	
Endometrial thickness on transfer day (mm)	10.47 ± 1.61	9.83 ± 1.89	10.39 ± 2.27	0.093
Number of embryos transferred				0.968
1	34.9%(15/43)	32.7%(36/110)	33.3%(16/48)	
2	65.1%(28/43)	67.3%(74/110)	66.7%(32/48)	
Clinical pregnancy rate (%)	23.3%(10/43)	40.9%(45/110)	29.2%(14/48)	0.081
Live birth rate (%)	16.3%(7/43)	27.3%(30/110)	22.9%(11/48)	0.352
Ectopic gestation rate (%)	0.0%(0/10)	2.2%(1/45)	0.0%(0/14)	0.763
Early miscarriage rate (%)	30.0%(3/10)	28.9%(13/45)	7.1%(1/14)	0.234

F1, LH < 1.45 IU/L; F2, 1.45 IU/L ≤ LH ≤ 4.19 IU/L; F3, LH > 4.19 IU/L. Normally distributed continuous data are presented as mean ± SD, and non-normally distributed data as median (P25, P75). *P < 0.05 compared with F1; **P < 0.01 compared with F1; #*P <* 0.05 compared with F2; ##*P <* 0.01 compared with F2. Clinical pregnancy and live birth rates are calculated per embryo transfer cycle. Rates for ectopic gestation and early miscarriage were calculated per clinical pregnancy. An asterisk (*) in the overall *P-*value column indicates statistical significance (*P <* 0.05) among the three groups. According to the World Health Organization (WHO) criteria, this table specifically presents data for the obese subgroup (BMI ≥ 30.0 kg/m^2^).

### 3.5 Binary logistic regression analysis

A dual-model binary logistic regression analysis was performed to evaluate the independent effect of trigger-day LH levels on the clinical pregnancy outcome of the first transfer. The low LH group (Group 1) served as the reference group. Based on the fully adjusted model (Model 2), which accounted for baseline, clinical, and cycle-specific embryo transfer characteristics, the results in the total population showed no statistically significant differences in the clinical pregnancy rate between the medium LH group (Group 2: aOR = 0.945, 95% CI: 0.649–1.376, P = 0.767), the high LH group (Group 3: aOR = 1.130, 95% CI: 0.820–1.557, P = 0.457), and the low LH group. This suggests that after comprehensively adjusting for confounders, trigger-day LH levels are not independently associated with clinical pregnancy outcomes in overweight and obese women (**Table 10**). Stratified analyses by transfer type yielded consistent results. In the fresh embryo transfer subgroup, no significant differences were observed compared to the reference group (A2: aOR = 0.658, 95% CI: 0.356–1.218, P = 0.183; A3: aOR = 0.829, 95% CI: 0.498–1.379, P = 0.470; **Table 11**). Similarly, no significant independent associations were found in the frozen-thawed embryo transfer (FET) subgroup (B2: aOR = 1.225, 95% CI: 0.746–2.010, P = 0.422; B3: aOR = 1.480, 95% CI: 0.965–2.271, P = 0.072; **Table 12**). Further stratified analyses by pathological type and BMI category reinforced these findings. No statistically significant differences in clinical pregnancy rates were detected across LH groups in the PCOS subgroup (C2: aOR = 0.755, P = 0.445; C3: aOR = 0.930, P = 0.799; **Table 13**), the non-PCOS subgroup (D2: aOR = 1.056, P = 0.814; D3: aOR = 1.209, P = 0.353; **Table 14**), the overweight subgroup (E2: aOR = 0.923, P = 0.702; E3: aOR = 0.988, P = 0.947; **Table 15**), or the obese subgroup (F2: aOR = 0.786, P = 0.658; F3: aOR = 1.865, P = 0.148; **Table 16**). Finally, in the sensitivity analysis treating the trigger-day LH strictly as a continuous variable, the fully adjusted multivariable logistic regression model similarly did not demonstrate a statistically significant linear association with clinical pregnancy (aOR = 1.015, 95% CI: 0.958–1.076, P = 0.610; **Supplementary Table 3**).

**Table 10:** Binary logistic regression analysis of clinical pregnancy for the total population.

Variables	aOR (95% CI)	*P-*value	aOR[95% CI]	*P-*value
	Model 1		Model 2	
Group1	reference		reference	
Group2	1.037 (0.731-1.473)	0.838	0.945 (0.649-1.376)	0.767
Group3	1.225 (0.909-1.650)	0.182	1.130 (0.820-1.557)	0.457
Female age	0.975 (0.933-1.018)	0.25	0.975 (0.930-1.022)	0.287
Male age	0.970 (0.937-1.004)	0.084	0.968 (0.932-1.006)	0.094
BMI	0.929 (0.884-0.976)	0.003*	0.941 (0.892-0.993)	0.027*
PCOS	0.733 (0.537-1.000)	0.05	0.843 (0.604-1.177)	0.315
hCG trigger alone	reference		reference	
GnRH agonist alone	1.012 (0.758-1.349)	0.938	1.142 (0.838-1.556)	0.399
Dual trigger	0.737 (0.306-1.775)	0.496	1.061 (0.416-2.704)	0.902
Fertilization method	0.949 (0.697-1.291)	0.739	0.923 (0.662-1.287)	0.638
Duration of infertility (years)	0.972 (0.932-1.015)	0.196	0.972 (0.929-1.016)	0.211
FSH	0.933 (0.878-0.992)	0.026*	0.932 (0.874-0.994)	0.031*
AFC	1.005 (0.995-1.015)	0.294	1.003 (0.993-1.014)	0.545
Endometrial thickness	–	–	1.055 (0.986-1.129)	0.12
Transfer of high-quality embryo (Yes vs. No)	–	–	5.125 (3.830-6.856)	<0.001*
Total number of transferred embryos	–	–	1.118 (0.842-1.484)	0.441

aOR, adjusted odds ratio; CI, confidence interval; BMI, body mass index; PCOS, polycystic ovary syndrome; FSH, follicle-stimulating hormone; AFC, antral follicle count.* *P <* 0.05 indicates statistical significance. Model 1 was adjusted for baseline and clinical characteristics, including female age, male age, BMI, PCOS status, trigger method, fertilization method, years of infertility, basal FSH, and AFC. Model 2 was fully adjusted. It included all variables in Model 1, with the further inclusion of embryo transfer characteristics as covariates: endometrial thickness, transfer of high-quality embryo, and total number of transferred embryos.

**Table 11:** Binary logistic regression analysis of clinical pregnancy for fresh embryo transfer cycles.

Variables	aOR (95% CI)	P-value	aOR (95% CI)	P-value
	Model 1		Model 2	
Group1	reference		reference	
Group2	0.783 (0.436–1.408)	0.414	0.658 (0.356–1.218)	0.183
Group3	0.969 (0.600–1.564)	0.897	0.829 (0.498–1.379)	0.47
Female age	1.031 (0.961–1.106)	0.395	1.022 (0.949–1.100)	0.564
Male age	0.919 (0.868–0.972)	0.003*	0.922 (0.867–0.979)	0.008*
BMI	0.977 (0.911–1.049)	0.521	0.995 (0.924–1.073)	0.904
PCOS (Yes vs. No)	0.767 (0.434–1.356)	0.361	0.828 (0.457–1.497)	0.531
hCG trigger alone	reference		reference	
Dual trigger	1.822 (1.033–3.215)	0.038*	1.814 (1.005–3.275)	0.048*
Fertilization method	1.287 (0.766–2.165)	0.341	1.251 (0.728–2.148)	0.417
Years of infertility	0.986 (0.920–1.057)	0.693	0.985 (0.917–1.058)	0.681
FSH	0.899 (0.820–0.986)	0.024*	0.900 (0.820–0.989)	0.028*
AFC	1.005 (0.986–1.024)	0.636	1.003 (0.983–1.023)	0.762
Endometrial thickness	–	–	0.998 (0.898–1.109)	0.965
Transfer of high-quality embryo (Yes vs. No)	–	–	2.989 (1.895–4.716)	<0.001*
Total number of transferred embryos	–	–	1.729 (1.014–2.949)	0.044*

aOR, adjusted odds ratio; CI, confidence interval; BMI, body mass index; PCOS, polycystic ovary syndrome; hCG, human chorionic gonadotropin; GnRH, gonadotropin-releasing hormone; FSH, follicle-stimulating hormone; AFC, antral follicle count.* *P <* 0.05 indicates statistical significance. Model 1 was adjusted for baseline and clinical characteristics, including female age, male age, BMI, PCOS status, trigger method, fertilization method, years of infertility, basal FSH, and AFC. Model 2 was fully adjusted. It included all variables in Model 1, with the further inclusion of embryo transfer characteristics as covariates: endometrial thickness, transfer of high-quality embryo, and total number of transferred embryos. In the Fresh ET subgroup, only one patient received GnRH agonist alone trigger; to ensure model stability (as fresh ET is generally avoided after GnRHa-only trigger), this single case was excluded. Consequently, the trigger methods adjusted in this model included only “hCG trigger alone” (reference) and “Dual trigger”.

**Table 12:** Binary logistic regression analysis of clinical pregnancy for frozen-thawed embryo transfer cycles.

Variables	aOR (95% CI)	*P-*value	aOR (95% CI)	*P-*value
	Model 1		Model 2	
Group1	reference		reference	
Group2	1.222 (0.780–1.916)	0.382	1.225 (0.746–2.010)	0.422
Group3	1.411 (0.956–2.082)	0.083	1.480 (0.965–2.271)	0.072
Female age	0.939 (0.886–0.995)	0.034*	0.941 (0.882–1.004)	0.065
Male age	1.008 (0.962–1.056)	0.744	1.010 (0.959–1.064)	0.703
BMI	0.886 (0.828–0.949)	0.001*	0.883 (0.818–0.954)	0.002*
PCOS	0.676 (0.460–0.994)	0.047*	0.792 (0.516–1.214)	0.284
hCG trigger alone	reference		reference	
GnRH agonist alone	0.799 (0.564–1.132)	0.207	1.023 (0.696–1.503)	0.909
Dual trigger	0.650 (0.264–1.598)	0.348	0.997 (0.376–2.641)	0.995
Fertilization method	0.810 (0.547–1.200)	0.294	0.801 (0.515–1.246)	0.325
Years of infertility	0.967 (0.915–1.023)	0.244	0.959 (0.902–1.019)	0.174
FSH	0.954 (0.878–1.038)	0.273	0.958 (0.875–1.050)	0.362
AFC	1.006 (0.994–1.018)	0.354	1.003 (0.990–1.017)	0.619
Endometrial thickness	–	–	1.100 (1.001–1.209)	0.047*
Transfer of high-quality embryo (Yes vs. No)	–	–	7.791 (5.221–11.627)	<0.001*
Total number of transferred embryos	–	–	0.913 (0.636–1.311)	0.622

aOR, adjusted odds ratio; CI, confidence interval; BMI, body mass index; hCG, human chorionic gonadotropin; GnRH, gonadotropin-releasing hormone; FSH, follicle-stimulating hormone; AFC, antral follicle count.* *P <* 0.05 indicates statistical significance.Model 1 was adjusted for baseline and clinical characteristics, including female age, male age, BMI, PCOS status, trigger method, fertilization method, years of infertility, basal FSH, and AFC. Model 2 was fully adjusted. It included all variables in Model 1, with the further inclusion of embryo transfer characteristics as covariates: endometrial thickness, transfer of high-quality embryo, and total number of transferred embryos.

**Table 13:** Binary logistic regression analysis of clinical pregnancy for PCOS patients.

Variables	aOR (95% CI)	*P-*value	aOR (95% CI)	*P-*value
	Model 1		Model 2	
Group1	reference		reference	
Group2	0.929 (0.477–1.808)	0.828	0.755 (0.368–1.551)	0.445
Group3	1.011 (0.602–1.697)	0.966	0.930 (0.532–1.626)	0.799
Female age	0.963 (0.880–1.053)	0.405	0.955 (0.867–1.051)	0.347
Male age	0.970 (0.892–1.055)	0.472	0.958 (0.875–1.048)	0.349
BMI	0.865 (0.790–0.947)	0.002*	0.893 (0.808–0.987)	0.027*
hCG trigger alone	reference		reference	
GnRH agonist alone	0.899 (0.553–1.462)	0.668	0.994 (0.586–1.688)	0.983
Dual trigger	0.314 (0.086–1.147)	0.08	0.427 (0.109–1.676)	0.223
Fertilization method	0.880 (0.458–1.692)	0.702	0.944 (0.462–1.926)	0.874
Years of infertility	0.979 (0.894–1.073)	0.651	0.977 (0.886–1.078)	0.645
FSH	0.981 (0.837–1.148)	0.807	0.916 (0.775–1.083)	0.303
AFC	1.006 (0.991–1.021)	0.424	1.003 (0.987–1.019)	0.723
Endometrial thickness	–	–	1.092 (0.951–1.254)	0.214
Transfer of high-quality embryo (Yes vs. No)	–	–	6.080 (3.374–10.956)	<0.001*
Total number of transferred embryos	–	–	1.211 (0.691–2.123)	0.503

aOR, adjusted odds ratio; CI, confidence interval; BMI, body mass index; hCG, human chorionic gonadotropin; GnRH, gonadotropin-releasing hormone; FSH, follicle-stimulating hormone; AFC, antral follicle count.* *P <* 0.05 indicates statistical significance.Model 1 was adjusted for baseline and clinical characteristics, including female age, male age, BMI,trigger method, fertilization method, years of infertility, basal FSH, and AFC. Model 2 was fully adjusted. It included all variables in Model 1, with the further inclusion of embryo transfer characteristics as covariates: endometrial thickness, transfer of high-quality embryo, and total number of transferred embryos.

**Table 14:** Binary logistic regression analysis of clinical pregnancy for non-PCOS patients.

Variables	aOR (95% CI)	*P-*value	aOR (95% CI)	*P-*value
	Model 1		Model 2	
Group1	reference		reference	
Group2	1.114 (0.730–1.700)	0.616	1.056 (0.671–1.660)	0.814
Group3	1.313 (0.906–1.904)	0.151	1.209 (0.810–1.804)	0.353
Female age	0.980 (0.931–1.032)	0.451	0.987 (0.934–1.043)	0.639
Male age	0.967 (0.930–1.005)	0.086	0.965 (0.926–1.007)	0.099
BMI	0.958 (0.905–1.015)	0.142	0.962 (0.903–1.024)	0.223
hCG trigger alone	reference		reference	
GnRH agonist alone	1.090 (0.757–1.570)	0.643	1.260 (0.853–1.861)	0.245
Dual trigger	1.646 (0.501–5.407)	0.411	2.599 (0.702–9.627)	0.153
Fertilization method	0.970 (0.682–1.379)	0.865	0.924 (0.633–1.349)	0.681
Years of infertility	0.971 (0.925–1.020)	0.244	0.974 (0.925–1.026)	0.323
FSH	0.921 (0.861–0.986)	0.018*	0.929 (0.866–0.997)	0.040*
AFC	1.004 (0.991–1.017)	0.555	1.003 (0.989–1.018)	0.67
Endometrial thickness	–	–	1.044 (0.965–1.130)	0.281
Transfer of high-quality embryo (Yes vs. No)	–	–	4.844 (3.448–6.804)	<0.001*
Total number of transferred embryos	–	–	1.150 (0.821–1.611)	0.417

aOR, adjusted odds ratio; CI, confidence interval; BMI, body mass index; hCG, human chorionic gonadotropin; GnRH, gonadotropin-releasing hormone; FSH, follicle-stimulating hormone; AFC, antral follicle count.* *P <* 0.05 indicates statistical significance.Model 1 was adjusted for baseline and clinical characteristics, including female age, male age, BMI, trigger method, fertilization method, years of infertility, basal FSH, and AFC. Model 2 was fully adjusted. It included all variables in Model 1, with the further inclusion of embryo transfer characteristics as covariates: endometrial thickness, transfer of high-quality embryo, and total number of transferred embryos.

**Table 15:** Binary logistic regression analysis of clinical pregnancy for overweight women.

Variables	aOR (95% CI)	*P-*value	aOR (95% CI)	*P-*value
	Model 1		Model 2	
Group1	reference		reference	
Group2	1.026 (0.699–1.506)	0.897	0.923 (0.611–1.394)	0.702
Group3	1.106 (0.796–1.537)	0.549	0.988 (0.693–1.408)	0.947
Female age	0.992 (0.944–1.044)	0.769	0.997 (0.945–1.052)	0.911
Male age	0.959 (0.922–0.998)	0.042*	0.953 (0.913–0.995)	0.028*
PCOS	0.680 (0.480–0.964)	0.030*	0.811 (0.558–1.179)	0.272
hCG trigger alone	reference		reference	
GnRH agonist alone	0.961 (0.698–1.323)	0.806	1.150 (0.817–1.621)	0.423
Dual trigger	0.522 (0.196–1.386)	0.192	0.799 (0.283–2.253)	0.672
Fertilization method	0.908 (0.646–1.278)	0.581	0.931 (0.644–1.345)	0.702
Years of infertility	0.964 (0.919–1.010)	0.122	0.959 (0.913–1.008)	0.103
FSH	0.906 (0.846–0.970)	0.005*	0.903 (0.841–0.969)	0.005*
AFC	1.008 (0.997–1.019)	0.153	1.005 (0.994–1.017)	0.375
Endometrial thickness	–	–	1.049 (0.973–1.131)	0.213
Transfer of high-quality embryo (Yes vs. No)	–	–	5.322 (3.836–7.382)	<0.001*
Total number of transferred embryos	–	–	1.033 (0.757–1.410)	0.839

aOR, adjusted odds ratio; CI, confidence interval; BMI, body mass index; PCOS, polycystic ovary syndrome; hCG, human chorionic gonadotropin; GnRH, gonadotropin-releasing hormone; FSH, follicle-stimulating hormone; AFC, antral follicle count.* *P <* 0.05 indicates statistical significance.Model 1 was adjusted for baseline and clinical characteristics, including female age, male age, PCOS status, trigger method, fertilization method, years of infertility, basal FSH, and AFC. Model 2 was fully adjusted. It included all variables in Model 1, with the further inclusion of embryo transfer characteristics as covariates: endometrial thickness, transfer of high-quality embryo, and total number of transferred embryos.

**Table 16:** Binary logistic regression analysis of clinical pregnancy for obese women.

Variables	aOR (95% CI)	*P-*value	aOR (95% CI)	*P-*value
	Model 1		Model 2	
Group1	reference		reference	
Group2	0.793 (0.295–2.132)	0.646	0.786 (0.271–2.283)	0.658
Group3	1.741 (0.806–3.762)	0.159	1.865 (0.801–4.342)	0.148
Female age	0.904 (0.818–0.999)	0.047*	0.887 (0.798–0.987)	0.028*
Male age	1.018 (0.940–1.103)	0.664	1.037 (0.949–1.133)	0.426
PCOS	0.996 (0.464–2.138)	0.993	1.036 (0.458–2.341)	0.933
hCG trigger alone	reference		reference	
Dual trigger	1.158 (0.553–2.425)	0.697	1.047 (0.467–2.351)	0.911
Fertilization method	1.102 (0.508–2.392)	0.806	0.921 (0.398–2.129)	0.847
Years of infertility	1.017 (0.909–1.138)	0.769	1.032 (0.914–1.165)	0.615
FSH	1.053 (0.899–1.234)	0.524	1.058 (0.890–1.258)	0.523
AFC	1.001 (0.977–1.025)	0.965	0.995 (0.968–1.022)	0.693
Endometrial thickness	–	–	1.052 (0.884–1.253)	0.565
Transfer of high-quality embryo (Yes vs. No)	–	–	4.075 (2.009–8.263)	<0.001*
Total number of transferred embryos	–	–	2.034 (0.928–4.456)	0.076

aOR, adjusted odds ratio; CI, confidence interval; BMI, body mass index; PCOS, polycystic ovary syndrome; hCG, human chorionic gonadotropin; GnRH, gonadotropin-releasing hormone; FSH, follicle-stimulating hormone; AFC, antral follicle count.* *P <* 0.05 indicates statistical significance.Model 1 was adjusted for baseline and clinical characteristics, including female age, male age, PCOS status, trigger method, fertilization method, years of infertility, basal FSH, and AFC. Model 2 was fully adjusted. It included all variables in Model 1, with the further inclusion of embryo transfer characteristics as covariates: endometrial thickness, transfer of high-quality embryo, and total number of transferred embryos. In the obese subgroup, only 3 patients received GnRH agonist alone trigger. To ensure the stability and validity of the multivariable logistic regression model, these 3 cases were excluded from the analysis. Consequently, the trigger methods adjusted in this model only included “hCG trigger alone” (reference) and “Dual trigger”.

### 3.6 Correlation analysis of serum LH level on the hCG trigger day

The study analyzed the correlation between serum LH level on the hCG trigger day and various clinical indicators. Before adjustment for multiple comparisons, serum LH level showed a weak positive correlation with basal FSH (r = 0.064, P = 0.031), and weak negative correlations with the number of follicles ≥14 mm on the trigger day (r = -0.156, *P <* 0.001), number of oocytes retrieved (r = -0.163, *P <* 0.001), number of mature oocytes (r = -0.173, *P <* 0.001), number of fertilized oocytes (r = -0.157, *P <* 0.001), number of normally fertilized oocytes (2PN) (r = -0.158, *P <* 0.001), number of transferable embryos (r = -0.106, *P <* 0.001), and number of high-quality embryos (r = -0.131, *P <* 0.001). No significant correlations were observed between serum LH level and female age, BMI, infertility duration, basal LH, basal E2, basal P, basal T, AFC, E2 on trigger day, or P on trigger day (all P > 0.05). After applying Bonferroni correction for the 18 variables tested (adjusted α = 0.05/18 = 0.0028), the correlation with basal FSH lost statistical significance (P = 0.031 > 0.0028), suggesting it may be a false positive finding. All correlations related to ovarian response and embryo quality remained statistically significant after correction (all *P <* 0.001). However, it should be noted that the absolute r_s_ values of these significant correlations ranged from 0.106 to 0.173, explaining less than 3% of the variance (r_s_^2^ < 0.03), indicating weak correlations. Although statistically significant due to the large sample size (n = 1,135), these associations have limited clinical relevance. Thus, with the increase of serum LH level on the hCG trigger day, the indicators related to ovarian reactivity and embryo quality show a weak downward trend, as shown in **Table 1**.

## 4 Discussion

Luteinizing hormone (LH) plays a pivotal role in regulating folliculogenesis and final oocyte maturation within a specific “LH window.” Suboptimal LH levels impair follicular energy metabolism and steroidogenesis, whereas abnormally elevated LH triggers premature luteinization and down-regulates LH receptors, ultimately compromising endometrial receptivity (8). Consequently, trigger-day serum LH levels serve as a critical reflection of the underlying endocrine and metabolic milieu.

While some studies report that elevated LH in GnRH antagonist protocols reduces oocyte yield without compromising final pregnancy rates (20–22), others indicate detrimental effects on live birth rates, particularly in vulnerable populations such as women with advanced maternal age (23) or diminished ovarian reserve (15).These conflicting findings suggest that the biological consequences of elevated LH are profoundly modulated by individual endocrine and metabolic phenotypes. However, overweight and obese populations—characterized by peripheral insulin resistance and HPOA axis disruption—have been largely overlooked. Given these unique metabolic derangements, extrapolating findings from the general population is insufficient. Therefore, this study independently evaluated the impact of trigger-day LH in overweight and obese women. A structured comparison of key methodological differences across studies is provided in **Supplementary Table 4**.

Our results demonstrated that elevated trigger-day LH was negatively correlated with oocyte yield and embryo quality. Although statistically significant, the absolute correlation coefficients were weak (explaining <3% of the variance), suggesting that elevated LH is not the sole direct cause of reduced embryo quality. Instead, elevated LH acts as a comprehensive manifestation of a “dual defect” inherent to women with elevated BMI (≥ 25 kg/m^2^). Neuroendocrinologically, these patients frequently exhibit HPOA dysfunction with accelerated LH pulse frequency. Metabolically, prevalent insulin resistance exacerbates hyperandrogenism, which synergistically disrupts granulosa cell function and weakens follicular responsiveness to gonadotropins (24, 25). Collectively, these intertwined pathways—neuroendocrine dysregulation and metabolic insulin resistance—render this overweight demographic inherently more susceptible to erratic LH elevations, which may trigger premature final follicular maturation. Consequently, clinical management often requires higher doses of exogenous gonadotropins to initiate follicular recruitment, inadvertently increasing treatment costs and potential risks such as ovarian hyperstimulation syndrome (OHSS). Notably, our study reveals distinct population heterogeneity regarding the detrimental effects of elevated LH. Subgroup analysis uncovered a key clinical phenomenon: in non-PCOS patients with BMI ≥ 25 kg/m^2^, the high-LH group was significantly associated with advanced maternal age (P = 0.007) and elevated basal FSH levels (P = 0.033). Mechanistically, these data suggest that abnormally high serum LH on the hCG trigger day in this specific cohort is more likely an endocrine manifestation of “early decline in ovarian reserve” or “physiological ovarian aging” (26).These patients typically exhibit poor ovarian response and require prolonged stimulation, leading to passive LH accumulation (27). In stark contrast, overweight PCOS patients exhibited a fundamentally different pathway. Stratified by LH, PCOS patients showed no significant differences in age, basal FSH, or antral follicle count (all P > 0.05), yet still exhibited a significant reduction in retrieved oocytes and transferable embryos. This divergence strongly indicates that the embryological decline in overweight PCOS women stems not from diminished ovarian reserve, but from a “synergistic toxicity” between the inherent pathophysiology of PCOS (e.g., endogenous LH hypersecretion) and severe insulin resistance (28).The sustained high LH exposure driven by this dual endocrine and metabolic disorder directly impairs the local ovarian microenvironment. This excessive LH exposure can induce premature follicular luteinization or compromise granulosa cell function, thereby exerting a direct inhibitory effect on follicular synchrony and oocyte quality, entirely independent of ovarian reserve (29).

Since their introduction into clinical practice in the late 1990s and early 2000s, GnRH antagonists have been widely used to inhibit pituitary activity, thereby preventing premature LH surges and premature ovulation before follicular maturation (30–32).Mechanistically, however, the precise impact of elevated LH on oocyte developmental competence remains a subject of debate. Some studies indicate that inappropriately high LH levels can negatively impact folliculogenesis by inhibiting FSH receptor expression, inducing follicular atresia, and reducing estradiol production (33). Furthermore, excessive LH may accelerate granulosa cell decline and disrupt the synchrony of oocyte maturation (16). However, LH receptors (LHR) are predominantly expressed in mural granulosa cells rather than cumulus cells, implying that elevated LH may not directly compromise the oocyte itself (34). Additionally, the chronic low-grade inflammatory state associated with obesity may weaken the regulatory efficacy of GnRH antagonists (35). These intertwined cellular mechanisms explain why population characteristics profoundly alter the clinical impact of LH fluctuations.

Despite the significant decline in embryological parameters, trigger-day LH was not an independent predictor of cumulative pregnancy or live birth rates. This aligns with findings in general GnRH antagonist cycles (36), though it contrasts with specific DOR cohorts (15). This discrepancy suggests that the clinical impact of elevated LH is likely context-dependent, relying heavily on patient-specific physiological characteristics rather than acting uniformly across all populations. Mechanistically, the lack of significant impact on pregnancy outcomes in our cohort may be partially explained by the progesterone profile. Aberrant LH elevations typically compromise pregnancy by triggering premature progesterone (P) rises, which interfere with endometrial receptivity (37). However, our data showed no significant variance in trigger-day progesterone levels across the three LH groups (P = 0.528), indicating that high LH levels did not induce severe premature luteinization prior to trigger. Collectively, these findings suggest that, at least in this overweight cohort, elevated LH alone is not the primary driver of impaired endometrial receptivity. Instead, elevated LH appears to exert its detrimental effect primarily by reducing the initial oocyte/embryo pool—acting as a mediator rather than a direct endometrial toxin. After adjusting for embryological and endometrial parameters (e.g., total transferable embryos, endometrial thickness) in our regression models, LH remained non-significant. As anticipated, established predictors such as female age, BMI, PCOS status, and basal FSH remained the primary drivers of pregnancy outcomes (38, 39).

Building on this, trigger-day LH demonstrated limited clinical utility in guiding embryo transfer strategies. Subgroup analyses revealed no significant independent effect on pregnancy outcomes in either fresh or frozen-thawed embryo transfer cycles (P>0.05). This indicates that while elevated LH compromises initial embryonic parameters, this detriment does not translate into differential clinical outcomes across transfer methods. Furthermore, even when considering the distinct endocrine profile inherent to PCOS, elevated trigger-day LH did not emerge as an independent risk factor for pregnancy outcomes in either the PCOS or non-PCOS cohorts.

To further explore the heterogeneity of LH effects across different BMI strata, we stratified our analysis by overweight (BMI 25–29.9 kg/m^2^) and obese (BMI ≥30 kg/m^2^) categories. Strikingly, while elevated trigger-day LH was associated with significantly reduced oocyte yield and embryo quality in the overweight cohort (all *P <* 0.05), these associations were completely absent in the obese group (all P > 0.05). Several mechanistic explanations may account for this intriguing observation. First, as BMI increases beyond 30 kg/m^2^, the reproductive endocrine milieu becomes increasingly dominated by profound insulin resistance, hyperinsulinemia, and chronic low-grade inflammation (25, 40).These metabolic disturbances may override or obscure the relatively modest contribution of LH to follicular development (3, 41).Second, the expression and function of the LH receptor (LHR) may be downregulated in the ovarian tissue of clinically obese individuals, potentially due to chronic exposure to inflammatory cytokines (42), thereby rendering granulosa cells less responsive to further LH elevation (43).Third, the higher prevalence of PCOS in the overweight high-LH group (33.6% vs 21.4%, P = 0.011) may partly drive the observed associations. In contrast, although the prevalence of PCOS in the obese cohort appeared to vary across the low-, medium-, and high-LH groups (27.9%, 35.5%, and 47.9%, respectively), this distribution did not reach statistical significance (P = 0.129), suggesting that different pathophysiological mechanisms operate at these distinct ends of the BMI spectrum. Clinically, these findings imply that while monitoring trigger-day LH may have prognostic value for embryological outcomes in overweight patients (BMI 25–29.9), its utility is limited in frankly obese patients (BMI ≥30), where metabolic factors likely play a more dominant role. Future studies with larger sample sizes in the obese category are warranted to validate these preliminary observations.

Regarding the primary outcome, our findings indicated that trigger day LH levels were not independently associated with clinical pregnancy rates. However, this null conclusion must be interpreted in the context of statistical power. Given our sample size distribution (e.g., n=272 in Group 1 and n=283 in Group 3) and an observed baseline pregnancy rate of approximately 50%, a *post-hoc* minimum detectable effect size (MDE) analysis revealed that our study was adequately powered (80% power, two-sided α=0.05) to detect an absolute difference in pregnancy rates of approximately 11.5% between groups. Therefore, we cannot rule out the possibility of a Type II error for smaller, subtle differences (e.g., <10%). Nevertheless, an absolute difference in pregnancy rates of less than 11.5% is arguably of limited clinical significance in routine practice. Thus, we conclude that trigger day LH is unlikely to have a clinically meaningful independent impact on pregnancy outcomes.

However, this study has several limitations. (1) Due to the retrospective and single-center design, causality cannot be definitively established. (2) We relied solely on BMI (≥25 kg/m^2^) without assessing body composition or metabolic syndrome criteria, meaning various metabolic phenotypes were pooled together despite potential differences in gonadotropin reactivity. (3) Our LH evaluation has methodological constraints: lack of standardized assay information, and sample-derived thresholds (1.45 and 4.19 IU/L) that require external validation. Although sensitivity analysis using LH as a continuous variable yielded consistent null results, we acknowledge that arbitrary trichotomization results in some information loss. Future studies using restricted cubic splines or established clinical thresholds (e.g., <1.2 or >5.0 IU/L) are needed. (4) Long-term neonatal outcomes were not evaluated. (5) We lacked longitudinal weight tracking; the interval between oocyte retrieval and frozen embryo transfers can span months or years, during which weight fluctuations may occur, introducing residual confounding. (6) Using WHO criteria may underestimate obesity in our population (Chinese guidelines define obesity as BMI ≥28 kg/m^2^), and the median BMI of our cohort exceeded this threshold.

## 5 Conclusions

For overweight infertile women, during COS, elevated serum LH level on the hCG trigger day is associated with impaired follicular development and decreased embryo quality, but no significant impact on pregnancy outcomes was observed. The above results suggest that in the GnRH antagonist protocol, the serum LH level on the hCG trigger day may affect embryological outcomes more than the final pregnancy outcomes. Therefore, during clinical controlled ovarian stimulation, it is necessary to comprehensively evaluate the individual characteristics of patients and reasonably adjust the gonadotropin dosage and medication regimen to maintain a relatively stable endocrine environment, thereby helping to obtain more ideal embryo quality.

## Data Availability

The original contributions presented in the study are included in the article/**Supplementary Material**, further inquiries can be directed to the corresponding author/s.

## References

[1] Liang Y Huang J Zhao Q Mo H Su Z Feng S . Global, regional, and national prevalence and trends of infertility among individuals of reproductive age (15–49 years) from 1990 to 2021, with projections to 2040. Hum Reprod. (2025) 40:529–44. doi: 10.1093/humrep/deae292. PMID: 39752330

[2] Buckett W Sierra S . The management of unexplained infertility: an evidence-based guideline from the Canadian Fertility and Andrology Society. Reprod BioMed Online. (2019) 39:633–40. doi: 10.1016/j.rbmo.2019.05.023. PMID: 31439397

[3] Broughton DE Moley KH . Obesity and female infertility: potential mediators of obesity’s impact. Fertil Steril. (2017) 107:840–7. doi: 10.1016/j.fertnstert.2017.01.017. PMID: 28292619

[4] Kudesia R Wu H Hunter Cohn K Tan L Lee JA Copperman AB . The effect of female body mass index on *in vitro* fertilization cycle outcomes: a multi-center analysis. J Assist Reprod Genet. (2018) 35:2013–23. doi: 10.1007/s10815-018-1290-6. PMID: 30132171 PMC6240553

[5] Liu D Li L Sun N Zhang X Yin P Zhang W . Effects of body mass index on IVF outcomes in different age groups. BMC Womens Health. (2023) 23:416. doi: 10.1186/s12905-023-02540-8. PMID: 37553621 PMC10410781

[6] Rafael F Rodrigues MD Bellver J Canelas-Pais M Garrido N Garcia-Velasco JA . The combined effect of BMI and age on ART outcomes. Hum Reprod. (2023) 38:886–94. doi: 10.1093/humrep/dead042. PMID: 36928306

[7] Balasch J Fábregues F . Is luteinizing hormone needed for optimal ovulation induction? Curr Opin Obstet Gynecol. (2002) 14:265–74. doi: 10.1097/00001703-200206000-00004. PMID: 12032381

[8] Pousias S Messini CI Anifandis G Sveronis G Georgoulias P Daponte A . The effect of a GnRH antagonist on follicle maturation in normal women. Reprod BioMed Online. (2019) 39:84–92. doi: 10.1016/j.rbmo.2019.03.100. PMID: 31129014

[9] Weghofer A Munné S Brannath W Chen S Barad D Cohen J . The impact of LH-containing gonadotropin stimulation on euploidy rates in preimplantation embryos: antagonist cycles. Fertil Steril. (2009) 92:937–42. doi: 10.1016/j.fertnstert.2008.07.1735. PMID: 18774557

[10] Luo Y Liu S Su H Hua L Ren H Liu M . Low serum LH levels during ovarian stimulation with GnRH antagonist protocol decrease the live birth rate after fresh embryo transfers but have no impact in freeze-all cycles. Front Endocrinol (Lausanne). (2021) 12:640047. doi: 10.3389/fendo.2021.640047. PMID: 33967956 PMC8104121

[11] Guo X Zhu X Wu X Yu Y Zhang L Shu J . Preventing growth stagnation and premature LH surge are the keys to obtaining a viable embryo in monofollicular IVF cycles: a retrospective cohort study. J Clin Med. (2022) 11(23):7140. doi: 10.3390/jcm11237140. PMID: 36498713 PMC9737977

[12] Zhou JS Chen JH Tang FF Ou JP Tao X Cai LH . The effect of luteinizing hormone changes in GnRH antagonist protocol on the outcome of controlled ovarian hyperstimulation and embryo transfer. BMC Pregnancy Childbirth. (2023) 23:604. doi: 10.1186/s12884-023-05916-8. PMID: 37612626 PMC10464317

[13] Segal L Fainaru O Kol S . Anovulatory patients demonstrate a sharp decline in LH levels upon GnRH antagonist administration during IVF cycles. Rambam Maimonides Med J. (2017) 8(2):e0021. doi: 10.5041/rmmj.10298. PMID: 28467764 PMC5415367

[14] Zhou R Dong M Huang L Zhu X Wei J Zhang Q . Association between serum LH levels on hCG trigger day and live birth rate after fresh embryo transfer with GnRH antagonist regimen in different populations. Front Endocrinol (Lausanne). (2023) 14:1191827. doi: 10.3389/fendo.2023.1191827. PMID: 37476498 PMC10354555

[15] Zhang Q Zhang K Gao Y He S Meng Y Ming L . Effect of LH level on HCG trigger day on clinical outcomes in patients with diminished ovarian reserve undergoing GnRH-antagonist protocol. Reprod Biol Endocrinol. (2024) 22:107. doi: 10.1186/s12958-024-01280-0. PMID: 39175038 PMC11340131

[16] Morimoto A Rose RD Smith KM Dinh DT Umehara T Winstanley YE . Granulosa cell metabolism at ovulation correlates with oocyte competence and is disrupted by obesity and aging. Hum Reprod. (2024) 39:2053–66. doi: 10.1093/humrep/deae154. PMID: 39013118 PMC11373349

[17] World Health Organization . Obesity: preventing and managing the global epidemic. Report of a WHO consultation. World Health Organ Tech Rep Ser. (2000) 894:i–xii, 1–253. doi: 10.1017/s0021932003245508. PMID: 11234459

[18] Alpha Scientists in Reproductive Medicine and ESHRE Special Interest Group of Embryology . The Istanbul consensus workshop on embryo assessment: proceedings of an expert meeting. Hum Reprod. (2011) 26:1270–83. doi: 10.1093/humrep/der037. PMID: 21502182

[19] Gardner DK Lane M Stevens J Schlenker T Schoolcraft WB . Blastocyst score affects implantation and pregnancy outcome: towards a single blastocyst transfer. Fertil Steril. (2000) 73:1155–8. doi: 10.1016/s0015-0282(00)00518-5. PMID: 10856474

[20] Kaur H Pranesh GT Rao V Rao KA . Effect of trigger day serum luteinising hormone levels on the in-vitro fertilization outcome: an observational study. Obstet Gynecol Sci. (2024) 67:235–42. doi: 10.5468/ogs.23215. PMID: 38325384 PMC10948215

[21] Huang Q Nong Y Zhang X Huang L Tang T Huang J . Effects of increasing serum luteinizing hormone levels during early phase of the gonadotropin-releasing hormone antagonist protocol on clinical outcomes of the *in vitro* fertilization cycle. Gynecol Endocrinol. (2022) 38:135–9. doi: 10.1080/09513590.2021.1955341. PMID: 34486905

[22] Geng Y Lai Q Xun Y Jin L . The effect of premature luteinizing hormone increases among high ovarian responders undergoing a gonadotropin-releasing hormone antagonist ovarian stimulation protocol. Int J Gynaecol Obstet. (2018) 142:97–103. doi: 10.1002/ijgo.12485. PMID: 29542120

[23] Gao F Wang Y Wu D Fu M Zhang Q Ren Y . A premature rise of luteinizing hormone is associated with a reduced cumulative live birth rate in patients ≥37 years old undergoing GnRH antagonist *In Vitro* fertilization cycles. Front Endocrinol (Lausanne). (2021) 12:722655. doi: 10.3389/fendo.2021.722655. PMID: 34925227 PMC8678590

[24] Legro RS . Obesity and PCOS: implications for diagnosis and treatment. Semin Reprod Med. (2012) 30:496–506. doi: 10.1055/s-0032-1328878. PMID: 23074008 PMC3649566

[25] Tang J Xu Y Wang Z Ji X Qiu Q Mai Z . Association between metabolic healthy obesity and female infertility: the national health and nutrition examination survey, 2013–2020. BMC Public Health. (2023) 23:1524. doi: 10.1186/s12889-023-16397-x. PMID: 37563562 PMC10416469

[26] Reichman DE Zakarin L Chao K Meyer L Davis OK Rosenwaks Z . Diminished ovarian reserve is the predominant risk factor for gonadotropin-releasing hormone antagonist failure resulting in breakthrough luteinizing hormone surges in *in vitro* fertilization cycles. Fertil Steril. (2014) 102:99–102. doi: 10.1016/j.fertnstert.2014.04.010. PMID: 24882557

[27] Conforti A Esteves SC Cimadomo D Vairelli A Di Rella F Ubaldi FM . Management of women with an unexpected low ovarian response to gonadotropin. Front Endocrinol (Lausanne). (2019) 10:387. doi: 10.3389/fendo.2019.00387. PMID: 31316461 PMC6610322

[28] Wang L Yu X Xiong D Leng M Liang M Li R . Hormonal and metabolic influences on outcomes in PCOS undergoing assisted reproduction: the role of BMI in fresh embryo transfers. BMC Pregnancy Childbirth. (2025) 25:368. doi: 10.1186/s12884-025-07480-9. PMID: 40155948 PMC11951658

[29] Qiao J Feng HL . Extra- and intra-ovarian factors in polycystic ovary syndrome: impact on oocyte maturation and embryo developmental competence. Hum Reprod Update. (2011) 17:17–33. doi: 10.1093/humupd/dmq032. PMID: 20639519 PMC3001338

[30] Felberbaum R Diedrich K . Ovarian stimulation for in-vitro fertilization/intracytoplasmic sperm injection with gonadotrophins and gonadotrophin-releasing hormone analogues: agonists and antagonists. Hum Reprod. (1999) 14:207–21. doi: 10.1093/humrep/14.suppl_1.207. PMID: 10573035

[31] Borm G Mannaerts B . Treatment with the gonadotrophin-releasing hormone antagonist ganirelix in women undergoing ovarian stimulation with recombinant follicle stimulating hormone is effective, safe and convenient: results of a controlled, randomized, multicentre trial. The European Orgalutran Study Group. Hum Reprod. (2000) 15:1490–8. doi: 10.1093/humrep/15.7.1490. PMID: 10875855

[32] Al-Inany HG Youssef MA Ayeleke RO Brown J Lam WS Broekmans FJ . Gonadotrophin-releasing hormone antagonists for assisted reproductive technology. Cochrane Database Syst Rev. (2016) 4:Cd001750. doi: 10.1002/14651858.CD001750.pub4. PMID: 27126581 PMC8626739

[33] Orisaka M Hattori K Fukuda S Mizutani T Miyamoto K Sato T . Dysregulation of ovarian follicular development in female rat: LH decreases FSH sensitivity during preantral-early antral transition. Endocrinology. (2013) 154:2870–80. doi: 10.1210/en.2012-2173. PMID: 23709086

[34] Assou S Anahory T Pantesco V Le Carroür T Pellestor F Klein B . The human cumulus--oocyte complex gene-expression profile. Hum Reprod. (2006) 21:1705–19. doi: 10.1093/humrep/del065. PMID: 16571642 PMC2377388

[35] Andreas E Winstanley YE Robker RL . Effect of obesity on the ovarian follicular environment and developmental competence of the oocyte. Curr Opin Endocr Metab Res. (2021) 18:152–8. doi: 10.1016/J.COEMR.2021.03.013. PMID: 38826717

[36] Doody K Devroey P Gordon K Witjes H Mannaerts B . LH concentrations do not correlate with pregnancy in rFSH/GnRH antagonist cycles. Reprod BioMed Online. (2010) 20:565–7. doi: 10.1016/j.rbmo.2009.12.019. PMID: 20133200

[37] Shi Q Jiang Y Kong N Huang C Liu J Shen X . Serum LH level on the day of hCG administration as a predictor of the reproductive outcomes in ovulation induction cycle frozen-thawed embryo transfer. J Pers Med. (2022) 13(1):52. doi: 10.3390/jpm13010052. PMID: 36675713 PMC9862278

[38] Shingshetty L Cameron NJ McLernon DJ Bhattacharya S . Predictors of success after *in vitro* fertilization. Fertil Steril. (2024) 121:742–51. doi: 10.1016/j.fertnstert.2024.03.003. PMID: 38492930

[39] Abdala A Elkhatib I Bayram A Arnanz A El-Damen A Melado L . Day 5 vs day 6 single euploid blastocyst frozen embryo transfers: which variables do have an impact on the clinical pregnancy rates? J Assist Reprod Genet. (2022) 39:379–88. doi: 10.1007/s10815-021-02380-1. PMID: 35064434 PMC8956773

[40] Szczesnowicz A Szeliga A Niwczyk O Bala G Meczekalski B . Do GLP-1 analogs have a place in the treatment of PCOS? New insights and promising therapies. J Clin Med. (2023) 12(18):5915. doi: 10.3390/jcm12185915. PMID: 37762856 PMC10532286

[41] Robker RL Akison LK Bennett BD Thrupp PN Chura LR Russell DL . Obese women exhibit differences in ovarian metabolites, hormones, and gene expression compared with moderate-weight women. J Clin Endocrinol Metab. (2009) 94:1533–40. doi: 10.1210/jc.2008-2648. PMID: 19223519

[42] Snider AP Wood JR . Obesity induces ovarian inflammation and reduces oocyte quality. Reproduction. (2019) 158:R79–90. doi: 10.1530/rep-18-0583. PMID: 30999278

[43] Menon KM Menon B . Regulation of luteinizing hormone receptor expression by an RNA binding protein: role of ERK signaling. Indian J Med Res. (2014) 140:S112–9. doi: 10.1074/jbc.m503154200. PMID: 25673531 PMC4345741

