# Supplementary material for: Impact of trigger-day serum luteinizing hormone levels on embryo quality and pregnancy outcomes in overweight and obese women undergoing GnRH antagonist protocols: a retrospective cohort study
Source: Front Endocrinol (Lausanne). 2026 May 8;17:1825688. doi: 10.3389/fendo.2026.1825688 (PMC13193990; doi:10.3389/fendo.2026.1825688)
Supplement: Supplementary file 1 [file DataSheet1.pdf]

Supplementary Table 1

Number of high-quality embryos	Group 1	Group 2	Group 3	*P*-value
**0 embryos**	**27.2% (74/272)**	**29.1% (169/580)**	**41.3% (117/283)**	**<0.001***
**1 embryo**	**27.9% (76/272)**	**31.6% (183/580)**	**25.8% (73/283)**	
**≥2 embryos**	**44.9% (122/272)**	**39.3% (228/580)**	**32.9% (93/283)**	

Supplementary Table 1 Distribution of the number of embryos among the three groups

Chi-square test was used to compare the distribution of the number of embryos among the three groups. $P < 0.05$ was considered statistically significant.
