# Supplementary material for: Impact of trigger-day serum luteinizing hormone levels on embryo quality and pregnancy outcomes in overweight and obese women undergoing GnRH antagonist protocols: a retrospective cohort study
Source: Front Endocrinol (Lausanne). 2026 May 8;17:1825688. doi: 10.3389/fendo.2026.1825688 (PMC13193990; doi:10.3389/fendo.2026.1825688)
Supplement: Supplementary file 2 [file DataSheet2.pdf]

Supplementary Table 2

Variables	aOR (95% CI)	*P*-value	aOR (95% CI)	*P*-value
	**Model 1**		**Model 2**	
Group1	reference		reference	
Group2	1.073 (0.710–1.621)	0.737	1.026 (0.661–1.592)	0.909
Group3	1.115 (0.783–1.587)	0.547	1.014 (0.695–1.479)	0.942
Female age	0.983 (0.934–1.034)	0.498	0.979 (0.927–1.034)	0.449
Male age	0.962 (0.924–1.002)	0.062	0.961 (0.920–1.003)	0.071
BMI	0.932 (0.879–0.989)	0.019*	0.936 (0.878–0.998)	0.043*
PCOS	0.757 (0.521–1.100)	0.145	0.908 (0.609–1.353)	0.635
Fertilization method	0.995 (0.698–1.418)	0.977	0.937 (0.641–1.370)	0.736
Years of infertility	0.970 (0.923–1.019)	0.23	0.975 (0.925–1.027)	0.342
FSH	0.927 (0.864–0.993)	0.032*	0.931 (0.866–1.001)	0.052
AFC	1.006 (0.994–1.019)	0.335	1.005 (0.992–1.019)	0.44
Endometrial thickness	-	-	1.032 (0.955–1.116)	0.42
Transfer of high-quality	-	-	4.277 (3.086–5.927)	<0.001*

**embryo (Yes vs. No)**				
**Total number of transferred embryos**	**-**	**-**	**1.326 (0.950–1.850)**	**0.097**

Supplementary Table 2 Binary logistic regression analysis of clinical pregnancy for patients receiving hCG trigger alone

Notes:aOR, adjusted odds ratio; CI, confidence interval; BMI, body mass index; PCOS, polycystic ovary syndrome; hCG, human chorionic gonadotropin; GnRH, gonadotropin-releasing hormone; FSH, follicle-stimulating hormone; AFC, antral follicle count.* $P < 0.05$ indicates statistical significance.Model 1 was adjusted for baseline and clinical characteristics, including female age, male age, BMI, PCOS status, trigger method, fertilization method, years of infertility, basal FSH, and AFC.Model 2 was fully adjusted. It included all variables in Model 1, with the further inclusion of embryo transfer characteristics as covariates: endometrial thickness, transfer of high-quality embryo, and total number of transferred embryos.
