# Supplementary material for: Impact of trigger-day serum luteinizing hormone levels on embryo quality and pregnancy outcomes in overweight and obese women undergoing GnRH antagonist protocols: a retrospective cohort study
Source: Front Endocrinol (Lausanne). 2026 May 8;17:1825688. doi: 10.3389/fendo.2026.1825688 (PMC13193990; doi:10.3389/fendo.2026.1825688)
Supplement: Supplementary file 3 [file DataSheet3.pdf]

Supplementary Table 3

**Variables**	**aOR (95% CI)**	***P*-value**	**aOR (95% CI)**	***P*-value**
	**Model 1**		**Model 2**	
**bLH**	**1.025 (0.971–1.082)**	**0.371**	**1.015 (0.958–1.076)**	**0.61**
**Female age**	**0.976 (0.934–1.020)**	**0.281**	**0.977 (0.932–1.024)**	**0.331**
**Male age**	**0.970 (0.937–1.004)**	**0.083**	**0.967 (0.932–1.004)**	**0.084**
**BMI**	**0.929 (0.885–0.977)**	**0.004***	**0.942 (0.893–0.994)**	**0.029***
**PCOS**	**0.776 (0.558–1.080)**	**0.132**	**0.865 (0.608–1.232)**	**0.423**
**hCG trigger alone**	**reference**		**reference**	
**GnRH agonist alone**	**1.020 (0.765–1.360)**	**0.894**	**1.147 (0.842–1.563)**	**0.383**
**Dual trigger**	**0.755 (0.313–1.819)**	**0.531**	**1.082 (0.423–2.764)**	**0.87**
**Fertilization method**	**0.941 (0.692–1.279)**	**0.696**	**0.918 (0.659–1.280)**	**0.615**
**Years of infertility**	**0.972 (0.932–1.014)**	**0.194**	**0.972 (0.929–1.017)**	**0.216**
**FSH**	**0.928 (0.874–0.986)**	**0.016***	**0.930 (0.872–0.991)**	**0.026***
**AFC**	**1.004 (0.994–1.014)**	**0.417**	**1.002 (0.992–1.013)**	**0.654**
**Endometrial thickness**	**-**	**-**	**1.053 (0.984–1.126)**	**0.137**

**Transfer of high-quality embryo (Yes vs. No)**	**-**	**-**	**5.132 (3.838–6.864)**	**<0.001***
**Total number of transferred embryos**	**-**	**-**	**1.117 (0.842–1.483)**	**0.442**

Supplementary Table 3 Binary logistic regression analysis of clinical pregnancy with LH as a continuous variable

Notes:aOR, adjusted odds ratio; CI, confidence interval; BMI, body mass index; PCOS, polycystic ovary syndrome; hCG, human chorionic gonadotropin; GnRH, gonadotropin-releasing hormone; FSH, follicle-stimulating hormone; AFC, antral follicle count.* $P < 0.05$ indicates statistical significance.Model 1 was adjusted for baseline and clinical characteristics, including female age, male age, BMI, PCOS status, trigger method, fertilization method, years of infertility, basal FSH, and AFC.Model 2 was fully adjusted. It included all variables in Model 1, with the further inclusion of embryo transfer characteristics as covariates: endometrial thickness, transfer of high-quality embryo, and total number of transferred embryos.
