# Supplementary material for: Impact of trigger-day serum luteinizing hormone levels on embryo quality and pregnancy outcomes in overweight and obese women undergoing GnRH antagonist protocols: a retrospective cohort study
Source: Front Endocrinol (Lausanne). 2026 May 8;17:1825688. doi: 10.3389/fendo.2026.1825688 (PMC13193990; doi:10.3389/fendo.2026.1825688)
Supplement: Supplementary file 4 [file DataSheet4.pdf]

Supplementary Table 4

Study / Reference	COS Protocol	Population Characteristics	Sample Size (cycles)	Timing of LH Measurement	Primary Outcome Definitions	LH Threshold Definitions	Main Findings Regarding Elevated Trigger-Day LH
Kaur et al., 2024 [20]	GnRH Antagonist	General infertile population	231	Morning of trigger day	Clinical pregnancy rate	<1.5, 1.5–3.0, > 3.0 IU/L	Reduced oocyte yield and mature oocytes; no significant impact on pregnancy outcomes.
Huang et al., 2022 [21]	GnRH Antagonist	General infertile population	4,204	Day of hCG administration	Clinical pregnancy and live birth rates	<1.2, 1.2–5.0, > 5.0 IU/L	Reduced number of retrieved oocytes; no detrimental effect on clinical pregnancy or live birth rates.
Geng et al., 2018 [22]	GnRH Antagonist	High ovarian responders	681	Day of hCG administration	Live birth rate	<1.0, 1.0-2.0, >2.0 IU/L	Did not negatively impact the live birth rate in high responders.

**Study / Reference**	**COS Protocol**	**Population Characteristics**	**Sample Size (cycles)**	**Timing of LH Measurement**	**Primary Outcome Definitions**	**LH Threshold Definitions**	**Main Findings Regarding Elevated Trigger-Day LH**
**Gao et al., 2021 [23]**	**GnRH Antagonist**	**Advanced maternal age (≥37 years)**	**902**	**Day of hCG administration**	**Cumulative live birth rate**	**Premature LH surge (>10 IU/L)**	**Significantly reduced cumulative live birth rates in older patients.**
**Zhang et al., 2024 [15]**	**GnRH Antagonist**	**Diminished Ovarian Reserve (DOR)**	**711**	**Morning of trigger day**	**Live birth rate**	**Cutoff at 4.29 IU/L (via ROC analysis)**	**Negatively affected embryological parameters and significantly reduced live birth rates in DOR patients.**
**Doody et al., 2010 [36]**	**GnRH Antagonist**	**General infertile population**	**1,501**	**Day of hCG administration**	**Clinical pregnancy rate**	**Continuous & categorical**	**No correlation between trigger-day LH levels and clinical pregnancy.**
**Current**	**GnRH Antag**	**Overweight and Obese**	**1,135**	**Morning of trigger**	**Cumulative clinical**	**<1.45, 1.45–4.19, >4.19**	**Reduced ovarian response**

Study / Reference	COS Protocol	Population Characteristics	Sample Size (cycles)	Timing of LH Measurement	Primary Outcome Definitions	LH Threshold Definitions	Main Findings Regarding Elevated Trigger-Day LH
Study	onist	women (BMI ≥25)		day	pregnancy and live birth rate	IU/L (percentiles)	and embryo quality; no independent effect on pregnancy outcomes.

Supplementary Table 4 Structured Comparison of Key Methodological Differences Among Cited Studies Evaluating Trigger-Day LH in GnRH Antagonist Protocols.
