# Supplementary material for: Impact of trigger-day serum luteinizing hormone levels on embryo quality and pregnancy outcomes in overweight and obese women undergoing GnRH antagonist protocols: a retrospective cohort study
Source: Front Endocrinol (Lausanne). 2026 May 8;17:1825688. doi: 10.3389/fendo.2026.1825688 (PMC13193990; doi:10.3389/fendo.2026.1825688)
Supplement: Supplementary file 5 [file DataSheet5.pdf]

Supplementary Table 5

Item No.	Recommendation	Section/Paragraph Reported
**Title and abstract**		
1(a)	Indicate the study's design with a commonly used term in the title or the abstract	Title; Abstract (Methods)
1(b)	Provide in the abstract an informative and balanced summary of what was done and what was found	Abstract
**Introduction**		
2	Explain the scientific background and rationale for the investigation being reported	Section 1 Introduction, Paragraphs 1-2
3	State specific objectives, including any prespecified hypotheses	Section 1 Introduction, Paragraph 3
**Methods**		
4	Present key elements of study design early in the paper	Section 2.1 Study population, Paragraph 1
5	Describe the setting, locations, and relevant dates, including periods of recruitment, exposure, follow-up, and data collection	Section 2.1 Study population, Paragraph 1
6(a)	Give the eligibility criteria, and the sources and methods of selection of participants. Describe methods of follow-up	Section 2.1.1 Inclusion/Exclusion Criteria; Section 2.2 Treatment Methods
6(b)	For matched studies, give matching criteria and number of exposed and unexposed	Not applicable
7	Clearly define all outcomes, exposures, predictors, potential confounders, and effect modifiers. Give diagnostic criteria, if applicable	Section 2.1.2 Grouping; Section 2.3 Outcome Measures
8	For each variable of interest, give sources of data and details of methods of assessment	Section 2.2 Treatment Methods; Section 2.3

	(measurement)	Outcome Measures
9	Describe any efforts to address potential sources of bias	Section 2.1.1 (Exclusion criteria); Section 2.4 (Adjustment via Model 1 and Model 2)
10	Explain how the study size was arrived at	Section 2.4 Statistical Analysis (Post-hoc power calculation)
11	Explain how quantitative variables were handled in the analyses. If applicable, describe which groupings were chosen and why	Section 2.1.2 Grouping (Categorization by P25 and P75); Section 2.4
12(a)	Describe all statistical methods, including those used to control for confounding	Section 2.4 Statistical Analysis
12(b)	Describe any methods used to examine subgroups and interactions	Section 2.1.2 (Subgroups); Section 2.4
12(c)	Explain how missing data were addressed	Section 2.1.1 (Exclusion criterion 6)
12(d)	If applicable, explain how loss to follow-up was addressed	Section 2.1.1 (Exclusion criterion 4 & 8)
12(e)	Describe any sensitivity analyses	Section 2.4; Section 3.5; Supplementary Table 3
Results		
13(a)	Report numbers of individuals at each stage of study—eg numbers potentially eligible, examined for eligibility, confirmed eligible, included in the study, completing follow-up, and analysed	Section 3.1; Figure 1
13(b)	Give reasons for non-participation at each stage	Figure 1
13(c)	Consider use of a flow diagram	Figure 1
14(a)	Give characteristics of study participants (eg demographic, clinical, social) and information	Section 3.1; Table 1; Tables

	on exposures and potential confounders	3-8
14(b)	Indicate number of participants with missing data for each variable of interest	Not applicable (Excluded cases with incomplete records)
14(c)	Summarise follow-up time (eg, average and total amount)	Section 2.2 Treatment Methods
15	Report numbers of outcome events or summary measures over time	Section 3.1; Table 2; Tables 3-8
16(a)	Give unadjusted estimates and, if applicable, confounder-adjusted estimates and their precision (eg, 95% confidence interval). Make clear which confounders were adjusted for and why they were included	Section 3.5; Tables 9-15
16(b)	Report category boundaries when continuous variables were categorized	Section 2.1.2 Grouping
16(c)	If relevant, consider translating estimates of relative risk into absolute risk for a meaningful time period	Not applicable
17	Report other analyses done—eg analyses of subgroups and interactions, and sensitivity analyses	Section 3.6; Table 16; Supplementary Tables 1-3
Discussion		
18	Summarise key results with reference to study objectives	Section 4 Discussion, Paragraphs 2-5
19	Discuss limitations of the study, taking into account sources of potential bias or imprecision. Discuss both direction and magnitude of any potential bias	Section 4 Discussion, Paragraph 7 (Limitations)
20	Give a cautious overall interpretation of results considering objectives, limitations, multiplicity of analyses, results from similar studies, and other relevant evidence	Section 4 Discussion, Paragraphs 2-6; Section 5 Conclusions
21	Discuss the generalisability (external validity) of the study results	Section 4 Discussion, Paragraph 7

**Other information**			
	**22**	**Give the source of funding and the role of the funders for the present study and, if applicable, for the original study on which the present article is based**	**Section 12 Funding**

Supplementary Table 5 STROBE Statement—Checklist of items that should be included in reports of cohort studies.

Notes: STROBE, Strengthening the Reporting of Observational Studies in Epidemiology. The reported page and line numbers correspond to the relevant sections in the revised manuscript where the recommended items are addressed.
